# Screening of Secretory Proteins Linking Major Depressive Disorder with Heart Failure Based on Comprehensive Bioinformatics Analysis and Machine Learning

**DOI:** 10.3390/biom14070793

**Published:** 2024-07-04

**Authors:** Chuanjing Zhang, Yongfei Song, Lichao Cen, Chen Huang, Jianqing Zhou, Jiangfang Lian

**Affiliations:** 1 Ningbo University Health Science Center, Ningbo 315040, China; 21818079@zju.edu.cn (C.Z.); lhlsongyongfei@nbu.edu.cn (Y.S.); 2101140096@nbu.edu.cn (L.C.); lhlzhoujianqing@nbu.edu.cn (J.Z.); 2 Department of Genetics, Xi’an Jiaotong University, Xi’an 710049, China; hchen@mail.xjtu.edu.cn

**Keywords:** major depressive disorder, heart failure, secretory protein, bioinformatics analysis, machine learning

## Abstract

Background: Major depressive disorder (MDD) plays a crucial role in the occurrence of heart failure (HF). This investigation was undertaken to explore the possible mechanism of MDD’s involvement in HF pathogenesis and identify candidate biomarkers for the diagnosis of MDD with HF. Methods: GWAS data for MDD and HF were collected, and Mendelian randomization (MR) analysis was performed to investigate the causal relationship between MDD and HF. Differential expression analysis (DEA) and WGCNA were used to detect HF key genes and MDD-associated secretory proteins. Protein–protein interaction (PPI), functional enrichment, and cMAP analysis were used to reveal potential mechanisms and drugs for MDD-related HF. Then, four machine learning (ML) algorithms (including GLM, RF, SVM, and XGB) were used to screen candidate biomarkers, construct diagnostic nomograms, and predict MDD-related HF. Furthermore, the MCPcounter algorithm was used to explore immune cell infiltration in HF, and MR analysis was performed to explore the causal effect of immunophenotypes on HF. Finally, the validation of the association of MDD with reduced left ventricular ejection fraction (LVEF) and the performance assessment of diagnostic biomarkers was accomplished based on animal models mimicking MDD. Results: The MR analysis showed that the MDD was linked to an increased risk of HF (OR = 1.129, *p* < 0.001). DEA combined with WGCNA and secretory protein gene set identified 315 HF key genes and 332 MDD-associated secretory proteins, respectively. Through PPI and MCODE analysis, 78 genes were pinpointed as MDD-related pathogenic genes for HF. The enrichment analysis revealed that these genes were predominantly enriched in immune and inflammatory regulation. Through four ML algorithms, two hub genes (ISLR/SFRP4) were identified as candidate HF biomarkers, and a nomogram was developed. ROC analysis showed that the AUC of the nomogram was higher than 0.90 in both the HF combined dataset and two external cohorts. In addition, an immune cell infiltration analysis revealed the immune dysregulation in HF, with ISLR/SFRP4 displaying notable associations with the infiltration of B cells, CD8 T cells, and fibroblasts. More importantly, animal experiments showed that the expression levels of ISLR (r = −0.653, *p* < 0.001) and SFRP4 (r = −0.476, *p* = 0.008) were significantly negatively correlated with LVEF. Conclusions: The MR analysis indicated that MDD is a risk factor for HF at the genetic level. Bioinformatics analysis and the ML results suggest that ISLR and SFRP4 have the potential to serve as diagnostic biomarkers for HF. Animal experiments showed a negative correlation between the serum levels of ISLR/SFRP4 and LVEF, emphasizing the need for additional clinical studies to elucidate their diagnostic value.

## 1. Introduction

Depression and heart failure (HF) both assume substantial roles in the worldwide disability burden [1]. Major depressive disorder (MDD), in particular, is widely recognized as a foremost mental health issue in modern society, representing perhaps the most debilitating and catastrophic psychiatric condition, with a considerable impact on 264 million individuals across the globe [2,3]. Conversely, HF emerges as a prevailing end-stage presentation of a multitude of cardiac conditions and has a profound and far-reaching impact on the overall well-being of patients [4].

In recent years, an accumulating body of evidence has highlighted the interdependence and complex relationships between MDD and HF. An observational cohort investigation involving 62,567 participants unveiled that patients with depression, particularly those diagnosed with MDD, faced a risk of HF that was 1.07 and 1.41 times higher, respectively, when compared to individuals without depression [5]. Numerous investigations have consistently yielded similar results [6,7]. Furthermore, a meta-analysis, encompassing a vast number of participants (80,627 individuals), has elucidated that MDD represents a notable independent risk factor and prognosticator for mortality and hospital readmission rates among individuals diagnosed with HF [8]. Interestingly, an additional investigation involving 204,523 individuals yielded findings indicating that a preceding history of MDD was associated with heightened all-cause mortality in the context of HF [9]. The aforementioned clinical trials collectively underscore the notion that the presence, or even a historical occurrence, of MDD may contribute to the initiation and progression of HF and consequently result in unfavorable clinical outcomes.

The precise mechanism by which MDD leads to the development of HF remains largely obscure. It is generally believed that the connection between mental diseases and peripheral organs is mainly related to the autonomic nervous system, neurohumoral processes, and the release of immune-inflammatory mediators [10]. These pathological processes all involve the release of secretory proteins and have been partially demonstrated. During depressive episodes, these proteins are secreted by brain tissues and blood cells and are subsequently transported to target organs through the circulatory system to confer their impacts [11].

Nevertheless, a significant gap exists in both fundamental research and clinical strategies regarding the manifestation of HF in the context of MDD. The early detection of HF in individuals with MDD represents an emerging and valuable research frontier that warrants attention and exploration [12].

Hence, this investigation used Mendelian randomization (MR) analysis to investigate the causal relationship between MDD and HF and employed an array of extensive bioinformatics tools to elucidate the hub genes and potential mechanisms of MDD-related HF based on secretory proteins. Furthermore, an advanced predictive diagnostic nomogram model for HF was developed through the utilization of five machine learning (ML) algorithms. In addition, the expression profiles of these hub genes were verified, and the diagnostic and predictive capabilities of the established nomogram were assessed using animal models of MDD.

## 2. Materials and Methods

### 2.1. MR Analysis of the Effect of MDD on HF

We conducted a two-sample MR analysis to investigate the causal relationship between MDD and HF. The summary data for MDD (exposure, ieu-a-1188) and HF (outcome, ebi-a-GCST009541) were obtained from the IEU OpenGWAS database (https://gwas.mrcieu.ac.uk/, accessed on 5 January 2024); the use of which has received informed consent from participants and has been granted ethical approval. These included 59,851 cases and 113,154 controls for MDD and 47,309 cases and 930,014 controls for HF.

#### 2.1.1. Instrumental Variable (IV) Selection Criteria

First, single-nucleotide polymorphisms (SNPs) that displayed genome-wide significance (*p* < 5 × 10^−6^) were selected from the MDD dataset. Second, to ensure the independence of SNPs, we conducted linkage disequilibrium (LD) clumping (r^2^ < 0.001, window size = 10,000 kb) to select independent significant SNPs. Third, the intensity of genetic variation was quantified by using an F-statistic. SNPs with F-statistic less than 10 were discarded, indicating that the intensity of genetic variation was insufficient. Following the above steps, the remaining SNPs were eventually used as IVs.

#### 2.1.2. MR Analysis

The standard variance inverse weighting (IVW) method was used as the main MR method. MR-Egger, weighted median, simple mode, and weighted mode methods are further complementary methods to MR. Cochran’s Q test was used to evaluate the heterogeneity among singular genetic variance estimations. When the *p*-value from Cochran’s Q test was below 0.05, we adopted a random effects model within IVW for the final MR analysis; otherwise, a fixed-effects model was selected [13].

#### 2.1.3. Sensitivity Analysis

The MR-Egger intercept term was used to evaluate the horizontal pleiotropy, and its *p* > 0.05 indicated the absence of horizontal pleiotropy. Heterogeneity was evaluated by Cochran’s Q test (*p* > 0.05), indicating no heterogeneity. The MR pleiotropy residual sum and outlier (MR-PRESSO) method was used to exclude horizontal multi-effect outliers that could have seriously affected the estimation results. The leave-one-out sensitivity analysis was conducted to eliminate each SNP in turn, so as to detect whether the SNP had an impact on the results. Furthermore, the MR Steiger directional test was employed to further assess the correlation between the exposure and the outcome [14].

The above MR analysis was conducted using the TwoSampleMR and MR-PRESSO packages in R (version 4.3.1). The full code used for MR and subsequent bioinformatics analysis can be found in Supplementary File S1.

### 2.2. Expression Data Collection and Processing

Three microarray datasets relating to the HF and control groups, namely GSE19303, GSE1145, and GSE21610, were retrieved from the GEO database (accessible at https://www.ncbi.nlm.nih.gov/geo/, accessed on 5 January 2024). The raw expression profile datasets of peripheral blood mononuclear cells (PBMCs) (GSE38206) and brain tissues (GSE80655) including anterior cingulate cortex (AnCg), dorsolateral prefrontal cortex (DLPFC), and nucleus accumbens (nAcc) from MDD patients were also obtained from GEO database as well. The integration of HF-related expression profiles was achieved through the application of the “ComBat” function of the “SVA” package in R (version 4.3.1). This process involved the batch correction of three HF-related datasets, resulting in a final compilation of 76 HF samples and 27 control samples. Detailed descriptive information on datasets and samples is shown in Table S1.

### 2.3. Differentially Expressed Genes (DEGs) Analysis

The HF combined dataset and MDD-related datasets underwent background correction, normalization, and gene symbol conversion. Subsequently, the identification of DEGs in HF- and MDD-related datasets was pinpointed through the utilization of the “Limma” and “DESeq2” packages in the R software program. Consequently, the screening of DEGs in HF- and MDD-related datasets was executed based on the criteria of adjusted *p*-value < 0.05 and |log2 (fold change)| > 0.5. Following this, the expression patterns of the DEGs were visually represented through the utilization of the “ggplot2” package for volcano plots and the “pheatmap” package for heatmaps in the R software program, respectively.

### 2.4. Weighted Gene Co-Expression Network Analysis (WGCNA) and Key Module Gene Identification

The utilization of WGCNA, recognized as a systematic biological methodology, aimed to disclose patterns of gene associations among various samples and to identify potential biomarker genes or therapeutic targets. The interplay among gene sets and their correlation with phenotypes assumed an integral role in the selection process. Step 1 involves removing genes with a median absolute deviation of 0 from each sample. In Step 2, the “goodSamplesGenes” function is utilized to identify missing values, and samples exceeding a cut height threshold of 20,000 are eliminated as outliers. In Step 3, an optimal soft threshold of 3 is determined using cex1 = 0.85, facilitating the construction of a scale-free co-expression gene network. Following gene clustering, modules are obtained, and similar ones are merged based on MEDissThres = 0.25 criteria. Step 4 entails generating a heat map illustrating module–trait relationships, while Step 5 involves calculating module membership (MM) and gene significance (GS) values and plotting scatter plots depicting MM-GS correlations for each module [11]. The specific codes for WGCNA can be found in Supplementary File S1.

### 2.5. Secretory Proteins Acquisition

The Human Protein Atlas database (accessible at https://www.proteinatlas.org/, accessed on 6 January 2024) provided access to download secretory proteins. The genes encoding secretory proteins, totaling 3947, were acquired from the class of “SPOCTOPUS-predicted secretory proteins” (https://www.proteinatlas.org/search/protein_class%3ASPOCTOPUS+predicted+secreted+proteins, accessed on 6 January 2024).

### 2.6. The Construction of Protein–Protein Interaction (PPI) Network

The exploration of interactions between MDD-associated secretory proteins and HF key genes involved the establishment of a PPI network. This network integrated MDD and HF based on data derived from the STRING database (https://www.string-db.org, accessed on 30 May 2024). A medium confidence score threshold exceeding 0.4 was applied in this context. Subsequently, the visualization of the PPI network was achieved through the utilization of the Cytoscape software (version 3.9.0). Furthermore, an additional step involved the implementation of the Cytoscape plug-in known as molecular complex detection (MCODE) for the identification of noteworthy subsets in the network. Subsets with scores greater than 10 were singled out as MDD-related pathogenic genes for further analytical procedures.

### 2.7. Functional Enrichment Analysis

The exploration of the biological function and mechanistic underpinnings of MDD-related pathogenic genes involved the application of Gene Ontology (GO) and Kyoto Encyclopedia of Genes and Genomes (KEGG) pathway enrichment analyses. The genes were imported into the Sangerbox database (http://www.sangerbox.com/tool, accessed on 30 May 2024) for this purpose. The enrichment significance was determined with a threshold set at *p* < 0.05. Furthermore, the findings of the functional enrichment analysis were visually represented through the utilization of a lollipop chart and a circos plot.

### 2.8. Connectivity Map (cMAP) Analysis

The cMAP, an online resource (https://clue.io, accessed on 30 May 2024), serves as a repository of gene expression profiles that operates by analyzing the impact of gene expression signatures, thereby elucidating connections between genes, diseases, and small-molecule compounds. According to official instructions, the up-regulated genes of cardiac origin in the MDD-related pathological genes were subsequently incorporated into the cMAP online database to unearth prospective small-molecule drugs for HF therapy. Ultimately, the study successfully pinpointed the top 10 compounds characterized by the most elevated enrichment scores.

### 2.9. ML Algorithms for the Screening of HF Biomarkers in MDD

For the identification of candidate biomarkers and establishment of a diagnostic model for HF, a combination of four different types of ML algorithms, including the random forest (RF), eXtreme Gradient Boosting (XGB), support vector machine (SVM), and generalized linear model (GLM), were employed in this research. We intersected MDD-associated secretory proteins with upregulated DEGs in HF and obtained 16 intersection genes. Then, four ML algorithms were used to perform diagnostic analysis of the intersected genes in the HF combined dataset, obtaining an importance score for each gene, and to rank the importance of each gene with a lollipop chart. In order to get the common important genes recognized by different algorithms, we intersect the top 5 genes scored by each of the four ML algorithms to obtain the hub genes. We used a violin diagram to show the expression pattern of hub genes and used a receiver operating characteristic (ROC) to analyze the diagnostic value of each hub gene for HF. The above four ML algorithms were carried out using the “glmnet”, “caret”, “random-Forest”, “kernlab”, and “xgboost” packages in R, and the detailed parameters of the ML algorithms for screening HF biomarkers are shown in Supplementary File S1.

### 2.10. The Construction and Assessment of Diagnostic Model for HF

The construction of the nomogram was achieved based on the two hub genes through the application of the “rms” package. The assessment of diagnostic performance for HF entailed the generation of ROC to examine the nomogram. In addition, calibration curves and a decision curve analysis (DCA) enabled the evaluation of the predictive efficiency of the nomogram in HF.

### 2.11. External Verification of Hub Genes Expression Pattern and Diagnostic Efficacy

Two independent datasets (GSE5406 and GSE141910) containing HF cases and controls were obtained from the GEO database. The expression patterns of hub genes in external datasets were analyzed by violin diagram, and the diagnostic effect of hub genes and nomogram model was evaluated by ROC analysis.

### 2.12. Immune Cell Infiltration Measurement

The quantification of immune cell and stromal cell infiltration from the gene expression profile of HF was performed using the “MCPcounter” package. The box plot, generated using the “ggplot2” package, was employed to present these significant differences. Following that, an examination of the connection between 10 subpopulations of immune cells was demonstrated using the “corrplot” package. Ultimately, Spearman’s rank correlation coefficient was employed to present the correlation of the expression of diagnostic biomarkers with the quantity of infiltrated immune cells.

### 2.13. MR Analysis of 731 Immune Cell Signatures on HF

We evaluated the causal relationship between 731 immune cell characteristics and HF based on a two-sample MR analysis. GWAS summary statistics for each immune trait are publicly available from the GWAS catalog (the entry numbers are from GCST0001391 to GCST0002121) [15]. The subsequent analysis process is referred to as MR analysis for MDD on HF.

### 2.14. In Vivo Experimental Protocols

The establishment of the chronic unpredictable mild stress (CUMS) model adhered to the methodologies delineated in prior research [16]. The CUMS molding time lasted 12 weeks, and the following environmental stress-inducing elements were applied: (1) the confinement of the subjects within small cages equipped with breathing holes; (2) the imposition of continuous overnight illumination; (3) the tilting of the cages to a 40° angle along the vertical axis for a duration of 24 h; (4) the reversal of the light/dark cycle for a duration of 24 h; (5) the introduction of damp bedding, consisting of a mixture of water (200 mL) and sawdust (100 g) for 24 h; and (6) the exposure to noise stimulation at a frequency of 60 Hz for a duration of 1 h. The application of the stressful stimulus occurred daily at 9:00 a.m., with a deliberate randomization of stimulus in order to prevent predictability by the animals. The control group of animals underwent an identical duration of housing in the absence of any form of stressful stimuli. Upon the culmination of the experiment, echocardiography was conducted, followed by the administration of anesthesia to euthanize the animals. Subsequently, serum and heart tissue samples were harvested for further experimental procedures.

### 2.15. Verification of the Expression of Hub Genes between Control and HF Groups

The extraction of total RNA from ventricular tissue was carried out utilizing the Trizol reagent (Vazyme, Nanjing, China). Thereafter, reverse transcription was carried out using a Reverse Transcription Kit (Vazyme), and a SYBR Green PCR Kit (Vazyme) was employed for the real-time quantitative PCR (RT-qPCR). Duplicate reactions were carried out to ensure accuracy, and the 2^−ΔΔCt^ approach was utilized for the quantitation of relative mRNA expression. The primer sequences are provided, including *ISLR* (F: 5′-CGGCCCCTCATTACTCCATC-3′; R: 5′-AACTCTCAAAGCGGGTCAGG-3′), *SFRP4* (F: 5′-TTTGGCAACGTACCTGAGCA-3′; R: 5′-CACTCCTCTGGACCGCTTTT-3′), and 18S rRNA (F: 5′-GAGAAACGGCTACCACATCC-3′; R: 5′-CACCAGACTTGCCCTCCA-3′). Moreover, the serum levels of ISLR and SFRP4 were validated using the designated ELISA kits (Bioswamp, Wuhan, China) in accordance with the guidelines provided by the manufacturer.

### 2.16. Statistical Analysis

To perform the statistical analysis, GraphPad Prism version 9.5.1 (GraphPad Software Inc., San Diego, CA, USA) was utilized in this study. Results were displayed as mean ± SD. The comparisons between the two groups were performed by means of an unpaired Student’s *t*-test. *p*-values < 0.05 were considered significant and significance for all figures determined as * *p* < 0.05, ** *p* < 0.01, *** *p* < 0.001, and **** *p* < 0.0001.

## 3. Result

### 3.1. MR Analysis of MDD on HF

**Figure 1:**
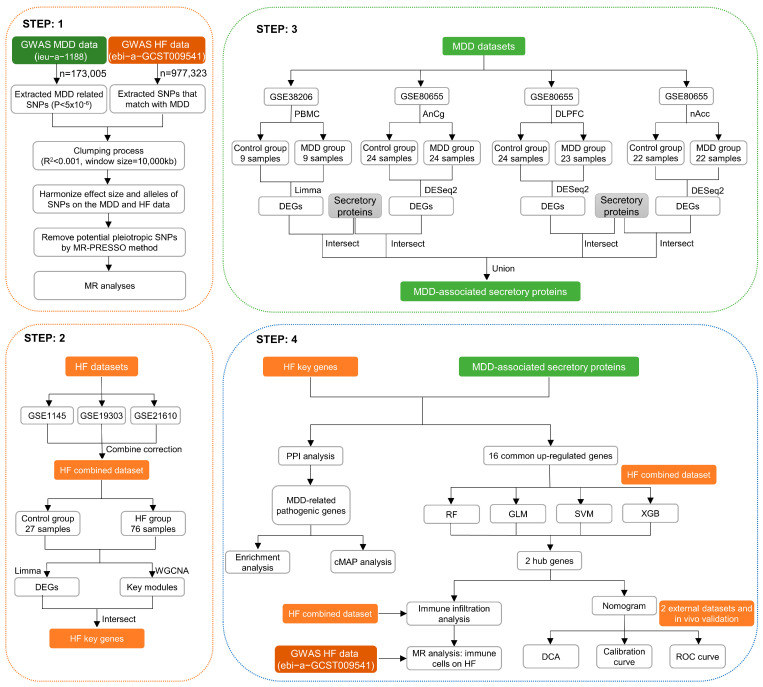
Graphical representation detailing the design flow of this research.

**Figure 2:**
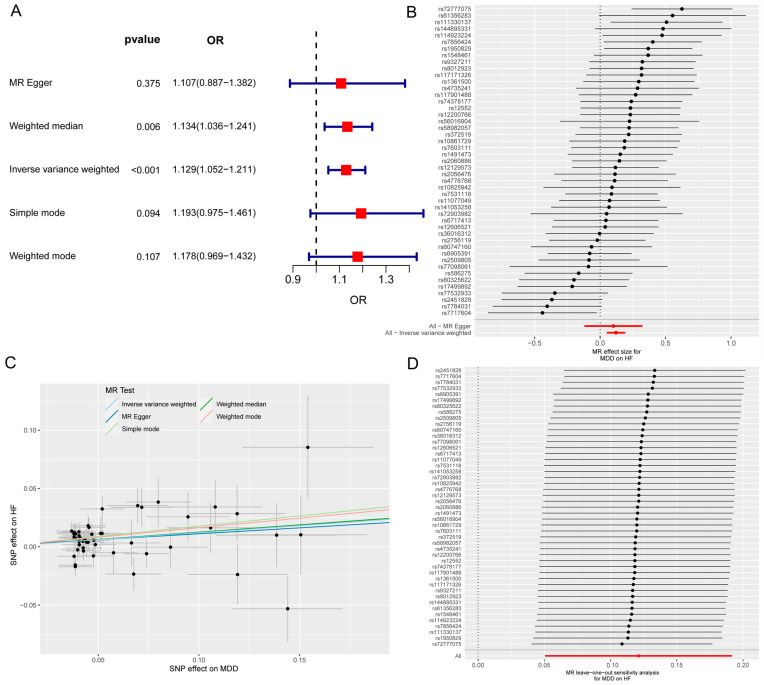
The MR analysis of MDD on HF. (**A**) Forest map shows MR analysis results for MDD and HF. (**B**) Forest map shows the MR effect size of each SNP for MDD on HF. (**C**) Scatter plot shows the effect of each SNP on MDD and HF. (**D**) Leave-one-out sensitivity analysis shows that the causal associations between MDD and HF are not affected by any individual SNP. MR, Mendelian randomization; MDD, major depressive disorder; HF, heart failure; SNP, single-nucleotide polymorphism.

The MR and bioinformatics analysis strategy is shown in Figure 1. The IVW analysis showed that MDD was linked to an increased risk of HF (OR, 1.129; 95% CI, 1.052–1211; *p* < 0.001) (Figure 2A). The effect size of each individual SNP is shown in Figure 2B,C, and the results of the leave-one-out sensitivity analysis (Figure 2D) suggested that the causal associations between MDD and HF were not affected by any individual SNP.

### 3.2. Identification of Differentially Expressed Genes in HF

**Figure 3:**
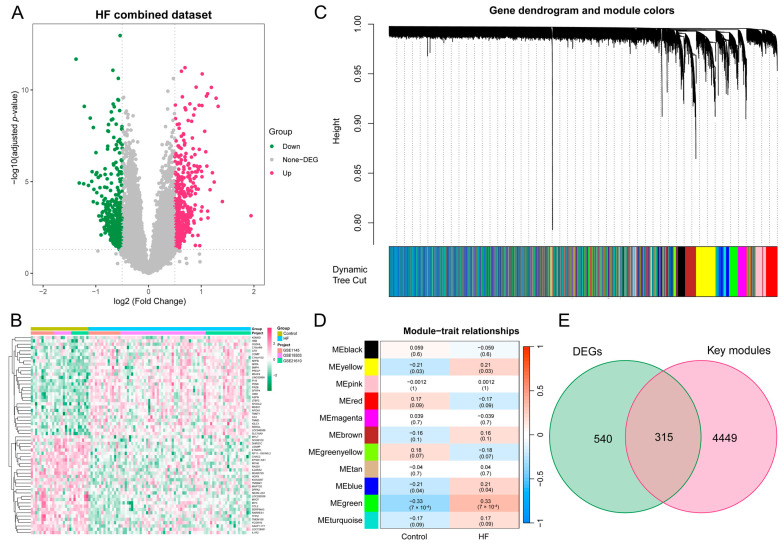
DEA and WGCNA of the HF combined dataset. (**A**) Volcano plot illustrating the DEGs in the HF combined dataset. In the visual representation, genes that are upregulated are symbolized by red dots, whereas genes that are downregulated are symbolized by green dots. (**B**) Heatmap featuring the top 30 upregulated and top 30 downregulated DEGs in the HF combined dataset. (**C**) Gene dendrogram displaying the cleaved gene modules. (**D**) Heatmap highlighting the associations of MEs with HF status, with green, yellow, and blue modules exhibiting significant correlations with HF (*p* < 0.05). (**E**) Venn diagram illustrating the identification of 315 HF key genes through the intersection of key modules with DEGs. DEA, differential expression analysis; WGCNA, weighted gene co-expression network analysis; HF, heart failure; DEG, differentially expressed gene; ME, module eigengene.

Subsequently, to batch correction, the HF combined dataset, comprising 76 samples from the HF group and 27 samples from the control group, was obtained. As illustrated in Figure S1A–D, the removal of batch effects led to a substantial reduction in differences observed among the three datasets. The DEA between the combined HF and control samples revealed a total of 855 DEGs, with 482 genes found to be upregulated and 373 genes downregulated. The expression pattern of these DEGs in the HF combined dataset is illustrated using a volcano plot and heatmap (Figure 3A,B).

### 3.3. The Construction of the Weighted Gene Co-Expression Network and the Identification of Key Modules in HF

To delve deeper into the identification of HF key genes, a WGCNA was performed to pinpoint the gene modules most closely linked to HF samples. The selection of the soft-thresholding power of 3, based on considerations of scale independence and average connectivity, is highlighted in Figure S2A. This power was applied to generate a total of 11 modules (Figure S2B), and the display of the clustering of module eigengenes can be observed in Figure 3C. Additionally, an investigation into the correlation between HF and 11 gene modules (Figure 3D) was carried out in this study. The analysis unveiled that the green module displayed the most positive correlation with HF (1146 genes, *p* = 7 × 10^−4^). The modules colored in green, yellow, and blue, which exhibited statistical significance, were designated as key modules for subsequent investigations. Moreover, we found a strong association between MM and GS in the green (r = 0.56, *p* = 1.4 × 10^−95^), blue (r = 0.33, *p* = 3.7 × 10^−59^), and yellow modules (r = 0.35, *p* = 9.9 × 10^−40^), respectively (Figure S2C–E). To identify key genes associated with HF, an intersection analysis was performed between DEGs and genes from WGCNA key modules. This process yielded a total of 315 HF key genes for further analysis (Figure 3E).

### 3.4. Identification of Differentially Expressed Secretory Proteins in MDD

**Figure 4:**
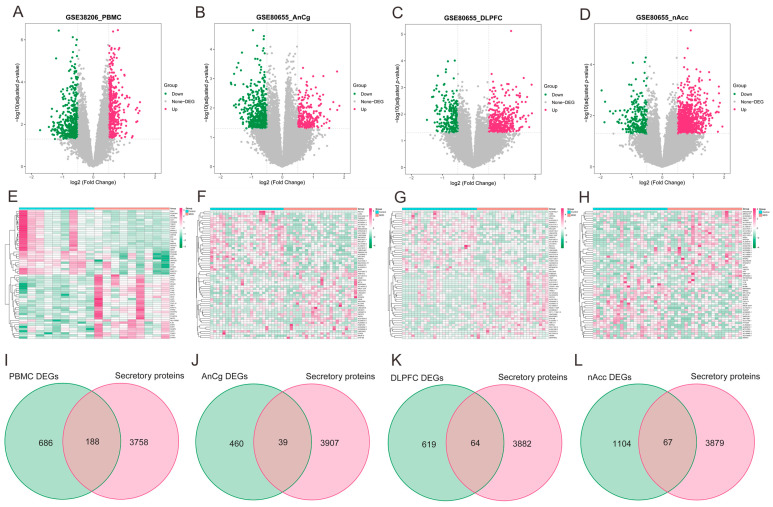
Identification of MDD-associated secretory proteins through DEA on secretory proteins in PMBCs and three brain area tissues related to MDD. (**A**–**D**) Volcano plots highlighting the DEGs in the MDD-PMBC dataset (**A**), MDD-AnCg dataset (**B**), MDD-DLPFC dataset (**C**), and MDD-nAcc dataset (**D**). (**E**–**H**) Heatmaps featuring the top upregulated (*n* = 30) and downregulated (*n* = 30) DEGs in the MDD-PMBC dataset (**E**), MDD-AnCg dataset (**G**), MDD-DLPFC dataset (**F**), and MDD-nAcc dataset (**H**). (**I**–**L**) Venn diagrams demonstrating the intersection of PBMC (**I**), AnCg (**J**), DLPFC (**K**), and nAcc (**L**) and genes-encoding secretory proteins, respectively. MDD, major depressive disorder; DEA, differential expression analysis; PBMC, peripheral blood mononuclear cells; AnCg, anterior cingulate cortex; DLPFC, dorsolateral prefrontal cortex; nAcc, nucleus accumbens; DEGs, differentially expressed genes.

As illustrated via volcano plots and heatmaps in Figure 4A–H, 874 DEGs were pinpointed in PBMCs of individuals with MDD, and in the regions of AnCg, DLPFC, and nAcc, the DEGs were found to be 499, 683, and 1171, respectively. Given the consideration that the development of HF may be primarily facilitated by the release of secretory proteins in individuals with MDD, the MDD-associated secretory proteins were subsequently acquired by combining the differentially expressed secretory proteins from MDD PBMC (Figure 4I) and brain tissue datasets (Figure 4J–L). This analysis collectively identified 332 MDD-associated secretory proteins.

### 3.5. Protein–Protein Interaction Network and Functional Enrichment and Drug Screening of the Pathogenic Genes Involved in MDD-Related HF

**Figure 5:**
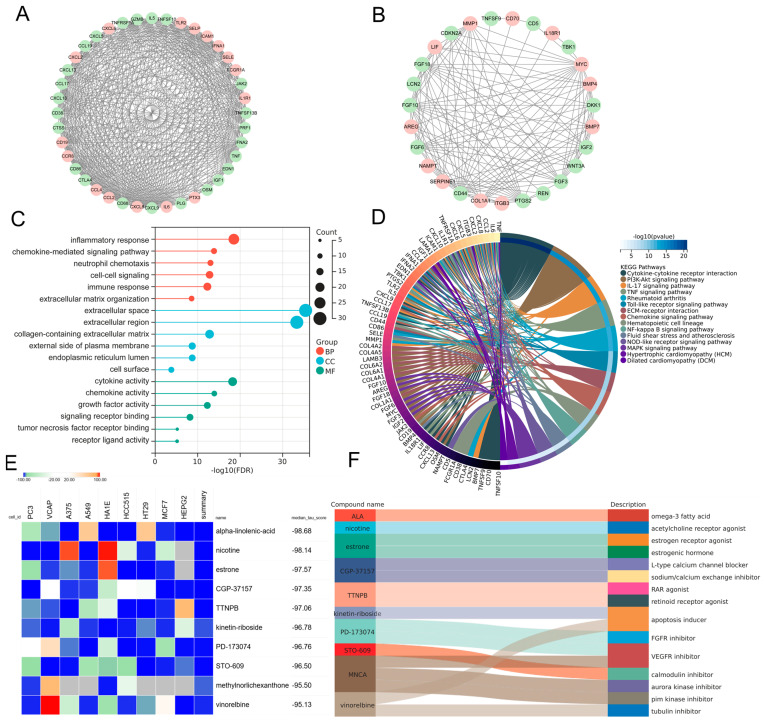
PPI analysis involving MDD-associated secretory proteins and HF key genes, followed by subsequent enrichment analysis and cMAP analysis of PPI-identified nodes. (**A**,**B**) PPI network of genes from subsets with the highest top-1 score (**A**) and top-2 score (**B**) as determined via Cytoscape plug-in MCODE analysis. In the PPI network, nodes colored in red represent genes that belong to the group of HF key genes, while green nodes represent genes that belong to the group of MDD-associated secretory proteins. (**C**) Lollipop chart highlighting the results of GO enrichment analysis covering BP, CC, and MF of MDD-related pathogenic genes. (**D**) Circos plot presenting the KEGG results pertaining to MDD-related pathogenic genes. (**E**) Heatmap presenting the top 10 compounds with the most significantly negative enrichment scores as indicated by the cMAP analysis. (**F**) The description of those top 10 compounds. PPI, protein–protein interaction; MDD, major depressive disorder; HF, heart failure; cMAP, connectivity map; MCODE, molecular complex detection; GO, gene ontology; BP, biological process; CC, cellular component; MF, molecular function; KEGG, Kyoto Encyclopedia of Genes and Genomes.

In order to elucidate the prospective pathogenic genes and mechanistic underpinnings in MDD-related HF, the interplay between MDD-associated secretory proteins and HF key genes was compiled using the STRING database. Through the utilization of the MCODE analysis plugin, subsets with scores greater than 10 were pinpointed, where 78 genes were highlighted as MDD-related pathogenic genes implicated in the development of HF (Figure 5A,B). The analysis of GO terms pertaining to biological process (BP) demonstrated that the MDD-related pathogenic genes exhibited a predominant enrichment in the “chemokine-mediated signaling pathway”, “inflammatory response”, and “extracellular matrix organization” (Figure 5C). The results of the molecular function (MF) analysis uncovered that the pathogenic genes displayed a strong association with “cytokine activity” and “chemokine activity” (Figure 5C). The findings of the KEGG pathway analysis unveiled robust associations between the MDD-related pathogenic genes and key pathways, namely “cytokine–cytokine receptor interaction” and “PI3K-Akt signaling pathway”. Additionally, these genes exhibited enrichment in fundamental factors contributing to HF, including “fluid shear stress and atherosclerosis”, “hypertrophic cardiomyopathy”, and “dilated cardiomyopathy” (Figure 5D). Furthermore, drug screening analysis of the cMAP database showed that the top 10 compounds with the highest negative scores, namely alpha-linolenic-acid (ALA), nicotine, estrone, CGP-37157, TTNPB, kinetin-riboside, PD-173074, STO-609, methylnorlichexanthone, and vinorelbine, were identified as potential pharmacological agents for the management of MDD-related HF (Figure 5E). The description of the targeted pathways pertaining to these compounds is manifested in Figure 5F.

### 3.6. Screening of Hub Genes Harboring Diagnostic Value via ML and Development of a Diagnostic Model for MDD-Related HF

**Figure 6:**
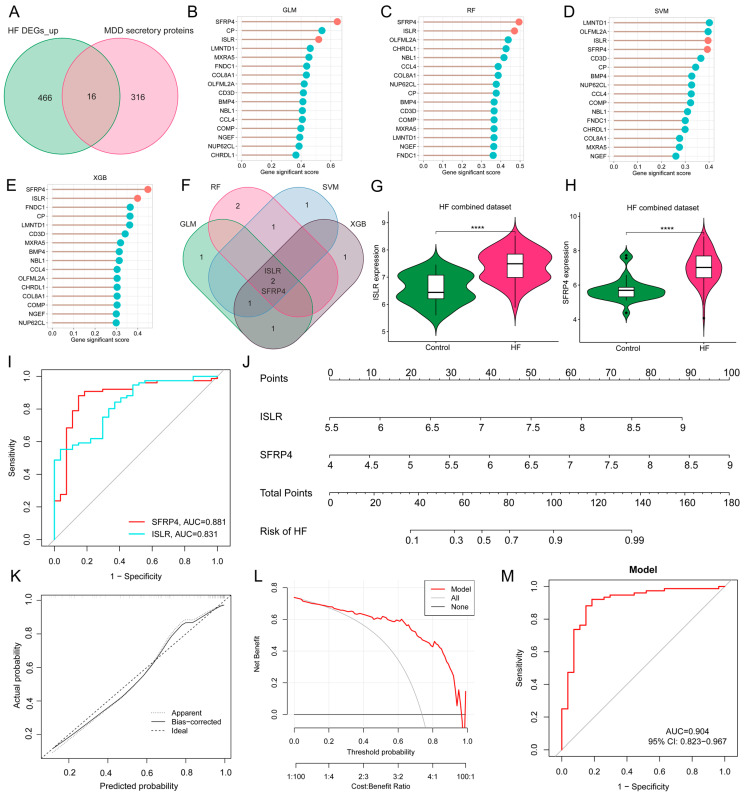
Screening of hub genes harboring diagnostic value via ML and development of a diagnostic model for MDD-related HF. (**A**) Venn diagram displaying the 16 intersecting genes among MDD-associated secretory proteins and up-regulated HF DEGs. (**B**–**E**) The lollipop charts show the importance ranking of 16 genes in GLM (**B**), RF (**C**), SVM (**D**), and XGB (**E**) algorithms. (**F**) Venn diagram revealing two common genes identified by all four ML algorithms. (**G**,**H**) The violin plot shows the expression patterns of ISLR (**G**) and SFRP4 (**H**) in the HF combined dataset. (**I**) ROC curve for the diagnostic performance of each candidate biomarker for HF. (**J**) Construction of a nomogram by considering the diagnostic biomarkers. (**K**) Calibration curve of the predictive performance of the nomogram model in MDD-related HF. The solid curve labeled “Bias-corrected” closely aligns with the dashed line labeled “Ideal” indicating the relative reliability of the results. (**L**) DCA for the performance of the nomogram model. The black line labeled as “None” represents the net benefit when assuming no HF patients, the grey line labeled as “All” indicates the net benefit when assuming all patients have HF, and the red line labeled as “Model” represents the net benefit based on the diagnostic predictions of nomogram model for HF in individuals with MDD comorbidity. (**M**) ROC curve for the diagnostic performance of the nomogram model (**M**) in the HF combined dataset. MDD, major depressive disorder; HF, heart failure; ML, machine learning; DEGs, differentially expressed genes; RF, random forest; SVM, support vector machine; XGB, eXtreme Gradient Boosting; GLM, generalized linear model; ROC, receiver operating characteristic; DCA, decision analysis curve.

Given the potential crucial involvement of common differentially expressed secretory proteins in MDD and HF, 16 common genes were identified at the intersection of MDD-associated secretory proteins and up-regulated HF DEGs (Figure 6A). To refine the selection of diagnostic biomarkers, four ML algorithms, namely GLM, SVM, RF, and XGB, were employed to assign rankings to the 16 common genes on the basis of the importance of each gene as a variable indicator (Figure 6B–E). It is worth noting that after the intersection of the top 5 important genes obtained by each of the four algorithms, only two hub genes were obtained, namely immunoglobulin superfamily containing leucine-rich repeat (ISLR) and secreted frizzled-related protein 4 (SFRP4) (Figure 6F). The violin plot shows the expression patterns of ISLR and SFRP4 in the HF combined dataset (Figure 6G,H), with ROC analysis showing that their AUC for HF diagnosis reached 0.831 and 0.881 (Figure 6I), respectively, suggesting their potential as HF biomarkers. To enhance the diagnostic accuracy and predictive capability, a nomogram was constructed incorporating the two hub genes (Figure 6J). The analysis of calibration curves unveiled a close resemblance between the predicted probabilities generated by the established nomogram diagnostic model and those of the ideal model (Figure 6K). Furthermore, the performance of the nomogram was assessed through DCA, revealing that the nomogram model-based decision-making could potentially offer advantages in the diagnosis of MDD-related HF (Figure 6L). In the ROC analysis, the AUC value of the nomogram model reaches 0.904 (Figure 6M).

### 3.7. External Verification of Hub Genes Expression Pattern and Diagnostic Efficacy

**Figure 7:**
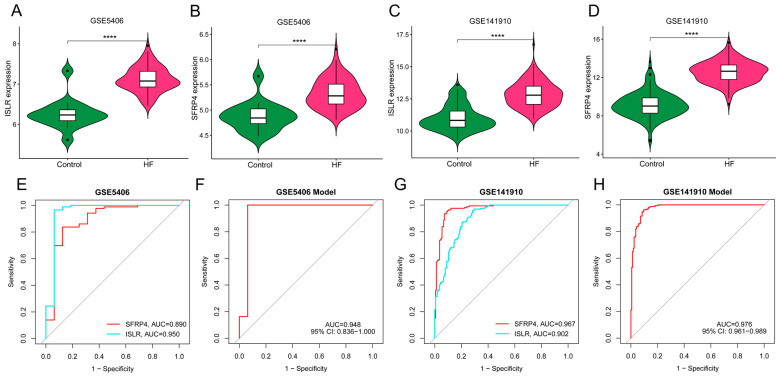
External verification of hub genes expression pattern and diagnostic efficacy. (**A**,**B**) The violin plots show the expression patterns of ISLR (**A**) and SFRP4 (**B**) in the GSE5406 dataset. (**C**,**D**) The violin plots show the expression patterns of ISLR (**C**) and SFRP4 (**D**) in the GSE141910 dataset. (**E**,**F**) ROC curve for the diagnostic performance of each candidate biomarker (**E**) and the nomogram model (**F**) for HF in the GSE5406 dataset. (**G**,**H**) ROC curve for the diagnostic performance of each candidate biomarker (**G**) and the nomogram model (**H**) for HF in the GSE141910 dataset. HF, heart failure; ROC, receiver operating characteristic.

We obtained two independent HF datasets (GSE5406 and GSE141910) from the GEO database. As shown in Figure 7A–D, ISLR and SFRP4 were significantly up-regulated in the HF group of both datasets. In the GSE5406 dataset, the AUC of SFRP4 and ISLR for HF diagnosis was 0.890 and 0.950, respectively (Figure 7E). In GSE141910, the AUC of SFRP4 and ISLR for HF diagnosis was 0.967 and 0.902, respectively (Figure 7G). In addition, the diagnostic efficiency of the nomogram model in GSE5406 and GSE141910 reached 0.948 and 0.976, respectively, suggesting that the combination of the two biomarkers has excellent diagnostic value.

### 3.8. Immune Cell Infiltration and MR Analysis of 731 Immune Cell Signatures on HF

**Figure 8:**
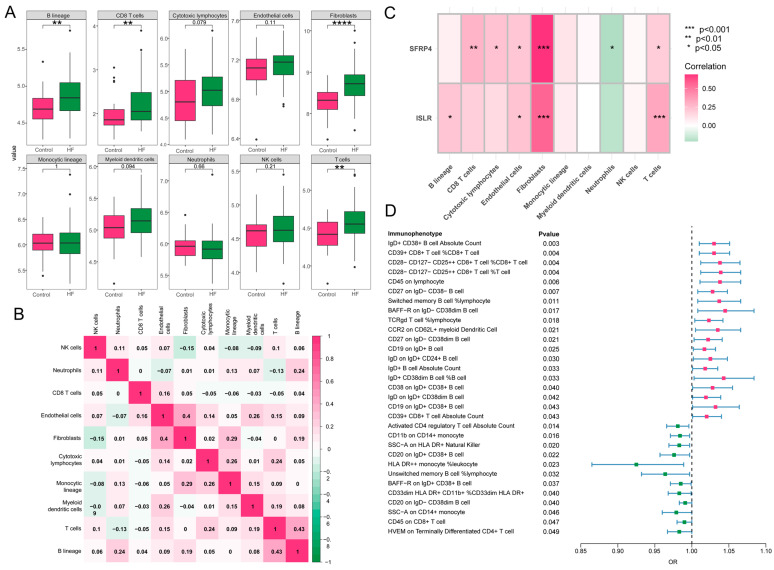
Immune cell infiltration and MR analysis in HF. (**A**) The box plot showing the differential comparison of the infiltration abundance of 10 immune-related cells between HF and control groups. (**B**) The heatmap reveals the correlation of infiltration of 10 immune-related cells. (**C**) The correlation plot represents the correlation between differentially infiltrated immune-related cells and two hub genes. (**D**) The forest map shows 31 immunophenotypes that are statistically significant in relation to the development of HF. MR, Mendelian randomization; HF, heart failure.

A close association was revealed between immune processes and the function and pathway analysis of MDD-related pathogenic genes in HF. The characteristics of eight subpopulations of immune cells and two subpopulations of stromal cells were derived using the MCPcounter algorithm. Higher proportions of B lineage, CD8 T cells, T cells, and fibroblasts were observed in HF in contrast to the control group (Figure 8A). Furthermore, a significant positive correlation (r = 0.43, *p* < 0.05) was found between T cells and B lineage during these 10 cell subpopulations, and fibroblasts demonstrated a positive association with endothelial cells (r = 0.4, *p* < 0.05) (Figure 8B). Notably, as displayed in Figure 8C, the hub genes, ISLR and SFRP4, showed a significant correlation to immune-related cell accumulation (especially T cells and fibroblasts) in HF. Additionally, the MR analysis showed that 31 immunophenotypes were associated with the development of HF, among which IgD+CD38+ B cells and CD39+CD8+ T cells were risk factors, and activated CD4 regulatory T cells were protective factors (Figure 8D).

### 3.9. Validation of the Expression Pattern of Two Hub Genes and Evaluation of the Diagnostic Value of the Nomogram Model

**Figure 9:**
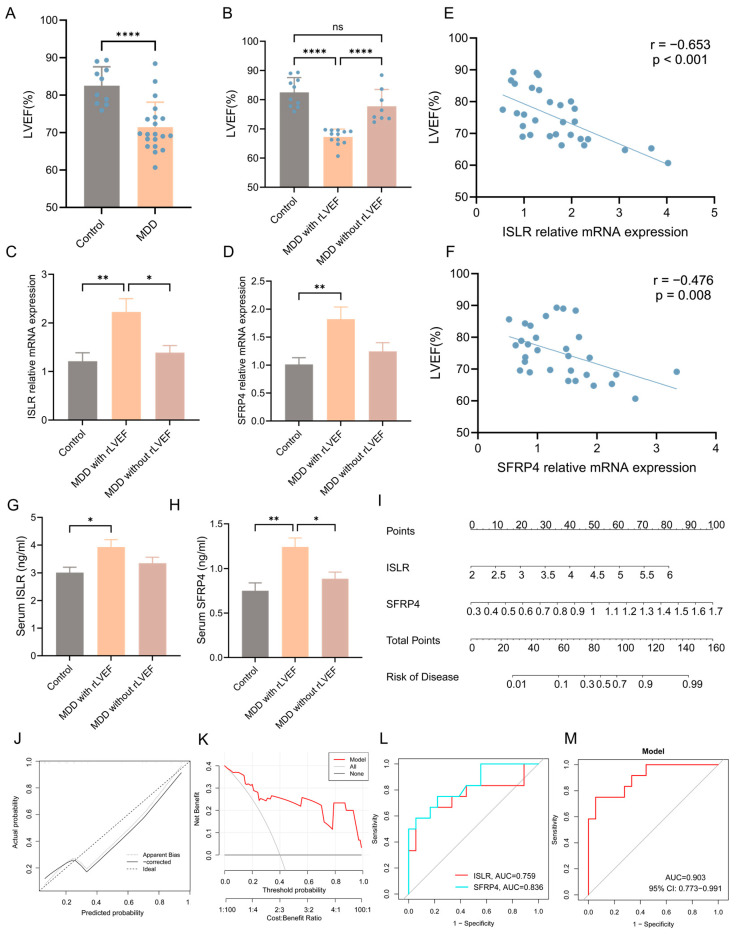
Validation of expression patterns of two hub genes and diagnostic performance of nomogram model in distinguishing HF using an MDD rat model. (**A**) Echocardiographic results indicating a notable decline in LVEF in the MDD group as compared to the control group. (**B**) Echocardiographic results indicating a reduction in LVEF in the MDD with rLVEF group compared to the control and MDD without rLVEF groups. (**C**,**D**) RT-qPCR findings highlighting an upregulation in the mRNA levels of *ISLR* (**C**) and *SFRP4* (**D**) in the left ventricular tissue of the MDD with rLVEF group. (**E**,**F**) Correlation analysis reflecting a negative correlation between the relative mRNA expression of *ISLR* (**E**) and *SFRP4* (**F**) in left ventricular tissue and LVEF. (**G**,**H**) ELISA results showing an elevation in the levels of ISLR (**G**) and SFRP4 (**H**) in the serum of rats in the MDD with rLVEF group as compared to the control and MDD without rLVEF groups. (**I**) Based on the expression levels of serum ISLR and SFRP4 in rats, a nomogram model based on the two biomarkers was established to predict the risk of MDD with rLVEF. (**J**,**K**) Calibration curve and DCA curve of nomogram model for predicting MDD with rLVEF. (**L**,**M**) ROC curve for the diagnostic performance of each candidate biomarker (**L**) and the nomogram model (**M**) constructed for MDD with rLVEF. HF, heart failure; MDD, major depressive disorder; rLVEF, reduced left ventricular ejection fraction; ELISA, enzyme-linked immunosorbent assay; DCA, decision analysis curve; ROC, receiver operating characteristic.

A well-recognized animal model of MDD (CUMS model) was established to enhance the validation of the integrated bioinformatics analysis described earlier. The echocardiographic results unveiled a notable reduction in left ventricular ejection fraction (LVEF) in the MDD group when compared to the control group, signifying decreased systolic function of the heart (Figure 9A). Nonetheless, it was observed that certain individuals in the MDD group did not manifest a reduction in LVEF. Consequently, the MDD group was stratified into two subgroups, namely the MDD with reduced LVEF (rLVEF) group and the MDD without rLVEF group on the basis of the mean LVEF value of rats in the MDD group. A notable observation was made wherein no significant difference in LVEF was evident between the MDD without rLVEF group and the control group. In contrast, rats in the MDD with rLVEF group displayed a marked reduction in LVEF compared with the control and MDD without rLVEF groups (Figure 9B). The findings from an RT-qPCR analysis provided confirmation that two hub genes in the MDD with rLVEF group exhibited an upregulation in the expression patterns in contrast to the control and MDD without rLVEF groups (Figure 9C,D). Subsequent correlation analysis unveiled a significant negative correlation between the mRNA expression of *ISLR* (r = −0.653, *p* < 0.001) and *SFRP4* (r = −0.476, *p* = 0.008) with LVEF (Figure 9E,F). In addition, the ELISA detection unveiled an elevated trend in the levels of ISLR and SFRP4 in the serum of the MDD with the rLVEF group when compared to non-HF rats (Figure 9G,H). A diagnostic nomogram model, incorporating ISLR and SFRP4, was developed for the prediction of the probability of rLVEF occurrence based on the in vivo experimental data (Figure 9I). The application of the nomogram in decision-making was found to potentially enhance the accuracy of rLVEF prediction, as indicated by the calibration curves and DCA results (Figure 9J,K). The ROC curve analysis unveiled that the AUC for ISLR and SFRP4 in diagnosing rLVEF reached 0.759 and 0.836, respectively. Notably, the AUC of the nomogram model reached 0.903, indicating its significantly advantageous diagnostic performance (Figure 9L,M).

## 4. Discussion

In this study, the relationship between MDD and subsequent HF was elucidated to some extent through the utilization of MR and a range of bioinformatics analysis methods. Potential mechanisms of MDD-related HF may include inflammatory and immune processes and signaling pathways such as “cytokine–cytokine receptor interaction” and “PI3K-Akt signaling pathway”. The utilization of ML algorithms facilitated the creation of a diagnostic nomogram model, incorporating two secretory hub genes, *ISLR* and *SFRP4*, to predict the risk of HF. The ROC curve analysis of our results demonstrated that these two hub genes exhibited excellent performance in predicting HF. Furthermore, the consistency of the upregulated expression patterns of *ISLR* and *SFRP4* with the obtained datasets was confirmed through the external validation of two GEO datasets and subsequent in vivo experimental verification. The serum levels of secretory proteins (ISLR and SFRP4) were further quantified. Subsequently, a diagnostic nomogram was developed, and the resulting model demonstrated remarkable predictive performance.

A mounting body of clinical research has indicated that individuals diagnosed with MDD experience a substantially heightened risk of HF [5], and we supported this association at the genetic level through MR analysis. Moreover, MDD and HF demonstrate a substantial intersection regarding psychosocial risk factors. Negative emotional states are frequently observed as a consequence of background factors such as political disparities, conflict, educational variations, economic differences, and discriminatory practices in society. These negative emotional states often result in unfavorable behaviors detrimental to cardiovascular function, such as inadequate dietary choices, smoking, excessive alcohol consumption, and insufficient physical activity. MDD imparts an inhibitory effect on self-care and diminishes the desire to engage with medical services and adhere to medication. These unfavorable lifestyle factors can affect the pathological causes, function aspects, and treatment outcomes of HF [10,17]. Nevertheless, certain investigations have demonstrated that, even after controlling for different adverse habits, MDD continues to exhibit a considerable association with a higher occurrence of HF, indicating a direct biological link between MDD and HF [18,19].

The specific mechanistic basis underlying the in vivo pathophysiological progression from MDD to HF remains largely unexplored. In the present research, the GO enrichment analysis of the MDD-related pathogenic genes unveiled that chemokine-mediated signaling pathway, inflammatory response, immune response, and extracellular matrix organization were the BPs with the highest enrichment scores. The migration of immune cells to the heart, which is widely acknowledged to assume a pivotal role in myocardial injury and repair, and inflammatory response and extracellular matrix organization, considered the core pathological process of HF, are well-established phenomena [20]. A subsequent KEGG enrichment analysis provided additional insights highlighting the enrichment of pathways such as the cytokine–cytokine receptor interaction, IL17 signaling pathway, and TNF signaling pathway. Prior research has documented that transgenic mice overexpressing TNF can stimulate left ventricular dilation and remodeling by modulating the activity of matrix metalloproteinases and activating the TGF-β pathway [21]. Furthermore, TNF has been recognized as an inflammatory marker in the context of HF [22]. The pro-inflammatory cytokine IL17 is implicated in HF through diverse pathways. Moreover, IL17 augments the apoptotic potential of mouse cardiomyocytes via the Stat3-iNOS pathway activation, leading to cardiac damage [23]. The upregulation of IL17 impairs cardiac functions through NF-kappa B-mediated disturbance of calcium regulation and cardiac remodeling [24]. These findings collectively suggest that immune and inflammatory responses triggered by MDD may be one of the mechanisms underlying the subsequent development of HF.

Currently, there is a paucity of clinical research on the treatment of HF co-occurring with MDD, typically necessitating separate treatment approaches for conventional HF and MDD. However, the efficacy of conventional HF therapeutics tends to diminish in individuals experiencing depressive states. Furthermore, the utilization of antidepressant drugs (such as tricyclic antidepressants and selective serotonin reuptake inhibitors) in the context of HF may not reduce the all-cause mortality of HF and could potentially even exacerbate this outcome [10]. Hence, there is an evident absence of specific recommendations pertaining to the choice of medications for HF management in patients with MDD. The present study provides a novel approach by establishing a connection between MDD-related pathogenic genes and the discovery of potential compounds targeting HF through the utilization of a cMAP analysis. Notably, ALA exhibited the most notable negative enrichment score in a cMAP analysis, meaning that it had the greatest potential for reversing the expression of upregulated MDD-related pathogenic genes in the context of HF. ALA is an essential omega-3 polyunsaturated fatty acid (ω-3PUFA) derived from plants that cannot be endogenously synthesized by the body and must be obtained through dietary sources. Current evidence strongly suggests that ω-3PUFA derived from fish, specifically eicosapentaenoic acid and docosahexaenoic acid, plays a significant role in preventing and managing HF by modulating triglyceride synthesis, enhancing mitochondrial function, and inhibiting arachidonic acid and TNF production. Conversely, research on the cardiovascular effects of plant-derived ALA remains limited and contentious. A cohort study involving 4432 participants did not identify a substantial correlation between dietary ALA consumption and the onset of HF through dietary analysis [25]. Conversely, a recent clinical study involving 905 HF patients, which measured serum phospholipids (a reflection of long-term ALA intake and metabolism), found that compared with patients with low serum phospholipid levels, patients with high phospholipid levels had statistically significant reductions in all-cause death, cardiac death, and first HF hospitalization [26]. Moreover, the findings from animal experiments indicate that a dietary intake of ALA for a duration of up to 12 months can effectively decrease the production of inflammatory factors (NF-κB and TNF), thereby postponing endothelial dysfunction and diastolic dysfunction in the cardiac tissue of aged mice [27]. These results provide evidence for the potential therapeutic use of ALA in the treatment of HF.

HF, in its early stages, often presents with insidious symptoms or solely as manifestations of the underlying disease. However, the onset of symptoms or an increase in levels of the HF-associated biomarker brain natriuretic peptide frequently indicates the transition of HF into the decompensation stage. Once this stage is reached, the progression of HF becomes nearly irreversible and typically leads to a terminal stage within a few years. Thus, HF contributes significantly to the global burden of disease [28]. Therefore, it is advantageous to detect and prevent HF at an early stage. This study utilized a range of ML algorithms to identify potential early indicators of HF in patients with MDD. Firstly, the GLM algorithm was employed due to its ability to accommodate categorical variables, extending the capabilities of traditional linear regression models by allowing response variables to adhere to a wider range of probability distributions [29]. Secondly, SVM classification was performed via the construction of an optimal hyperplane in the feature space. The advantage of SVM is that it can deal with complex problems in high-dimensional space, and it can also perform well for small training sets, especially for the binary classification problem of diagnosis in this study. In addition, integrated ML algorithms are combinations of multiple weak learning algorithms that can improve the performance of a single algorithm, among which RF and XGB are commonly used in the field of bioinformatics. RF is commonly utilized to enhance generalization performance and mitigate overfitting, while XGB is typically employed to improve training accuracy and prevent underfitting [30]. Therefore, we selected the above four ML algorithms to score the genetic importance of the HF combined dataset to screen out potential candidate biomarkers (ISLR and SFRP4). As verified by external data sets and animal experiments, the high expression of ISLR and SFRP4 is significantly correlated with the occurrence of HF and has a high predictive value for the diagnosis of HF.

Most notably, this study further established a comprehensive diagnostic nomogram model based on two hub genes for the diagnosis of HF, and this model has been validated externally. In addition, our in vivo experiments highlighted that sustained MDD might result in mild-to-moderate cardiac systolic dysfunction. An analysis of the mRNA levels in heart tissue of the MDD with rLVEF group indicated a significantly higher expression of ISLR and SFRP4 than that of the control and MDD without rLVEF groups, thus corroborating the findings extracted from the GEO dataset. Notably, the development of a diagnostic nomogram model substantiated the effectiveness in accurately distinguishing the occurrence of rLVEF. ISLR serves as a marker for mesenchymal stromal cells. In murine models of myocardial infarction and stress overload, fibroblasts expressing ISLR exhibited a notable increase in proliferation. Conversely, mice lacking ISLR displayed marked myocardial fibrosis, indicating a crucial role for ISLR in the repair of myocardial damage and fibrosis. SFRP4 exhibits elevated expression levels in the heart tissue of HF individuals, and the inhibition of SFRP4 has been shown to mitigate mitochondrial dysfunction, apoptosis of myocardial tissue, and interstitial fibrosis. Furthermore, SFRP4 functions as a high-affinity protein capable of binding to Wnt ligands, thereby playing a crucial role in various pathological processes associated with HF, including myocardial hypertrophy and fibrosis, through the modulation of Wnt pathway activity. The aforementioned evidence suggests that ISLR and SFRP4 may serve as potential biomarkers for HF in patients with MDD, enabling healthcare providers to detect HF at an early stage and implement timely interventions to enhance patients’ quality of life.

During our examination of immune cell infiltration, we observed a significant recruitment of T cells, B cells, CD8 T cells, and fibroblasts in the failing heart. These findings align with prior experimental investigations in this area. Two weeks after transverse aortic constriction (TAC) in mice, there was a discernible recruitment of T cells towards the cardiac tissue. Following this recruitment, a noteworthy increase in the abundance of CD8 T cells was noted 4 weeks after the TAC procedure. The kinetics of T-cell infiltration in the left ventricle (LV) was tightly linked to the progression of systolic dysfunction. Notably, in mice lacking T cells (TCRα−/−) subjected to TAC, LV systolic and diastolic functions were preserved; LV fibrosis, hypertrophy, and inflammation were mitigated; and the survival rate was enhanced compared to the control mice [31,32,33]. The role of B cells has been relatively poorly studied, but there are several lines of evidence suggesting that it plays an important role in myocardial injury. Specifically, the population of B cells in the myocardium escalates following acute cardiac injury in mice, and an investigation on B-cell-deficient mice demonstrated a protective effect against adverse LV remodeling subsequent to acute myocardial injury [34]. In addition, antibody-mediated B-cell depletion can alleviate angiotensin II-induced cardiomyocyte hypertrophy and collagen deposition [35]. Furthermore, additional compelling evidence supporting the involvement of B cells in HF lies in the detection of multiple autoantibodies targeting cardiac proteins in patients with dilated cardiomyopathy. These autoantibodies specifically target proteins such as the M2 muscarinic receptor, the cardiac troponin I, the β1-adrenergic receptor, the cardiac myosin, and the adenine nucleotide translocator [36]. Furthermore, MR analysis based on 731 immune cell signatures demonstrated the specific immunophenotypes leading to the occurrence of HF, such as IgD+CD38+ B cells, CD39+CD8+ T cells, and CD28-CD127-CD25+CD8+ T cells, providing a new direction for follow-up research.

In addition, the hub genes *ISLR* and *SFRP4* exhibited a strong association with the infiltration of immune cells and fibroblasts in HF. Consequently, it can be inferred that candidate biomarkers have the capability to not only distinguish HF from other conditions but also contribute to its progression by engaging with immune pathways. Therefore, it becomes imperative to achieve a comprehensive understanding of the immune pathways associated with HF to develop novel diagnostic or prognostic biomarkers and therapeutic targets for HF.

One of the strengths of this study is that we identified a decline in cardiac systolic function caused by persistent MDD in the CUMS rat model and found that serum ISLR/SFRP4 levels were negatively correlated with LVEF, which could rule out other comorbidities more easily than clinical studies. However, a limitation of this study is that no significant manifestations of HF were observed in the CUMS rat model at 3 months, which is insufficient to demonstrate the practical value of ISLR/SFRP4 as biomarkers of HF. Therefore, longer or more intense modeling of MDD, such as bondage stress or social failure stress, may be better animal models for studying MDD-induced HF. Furthermore, this study lacks clinical serum sample evidence, and rigorous clinical studies with large samples are needed to further prove the predictive efficacy of ISLR/SFRP4 as biomarkers of HF in MDD patients. Another finding of this study is that the potential mechanisms and therapeutic agents for the MDD induction of HF are revealed through MDD-associated secretory proteins, and further studies of relevant mechanisms and therapeutic targets are needed in vivo and in vitro. Finally, another limitation of this study is that we used GWAS summary data when performing an MR analysis. Summary-data-based analyses can be biased not only by errors in the GWAS and LD reference data sets but also by differences between them due to errors in GWAS resulting from data generation. Therefore, an MR analysis based on raw data may more accurately reflect the impact of MDD on HF.

## Data Availability

The public datasets were downloaded and analyzed in this study, including IEU OpenGWAS database (accession numbers: ieu-a-1188 and ebi-a-GCST009541) and GEO data repository (accession numbers: GSE1145, GSE19303, GSE21610, GSE38206, GSE80655, GSE5406, and GSE141910). Informed consent was obtained from all subjects involved in the study. All used source codes of MR and bioinformatics analysis are shown in Supplementary File S1.

## Supplementary Materials

The following supporting information can be downloaded at https://www.mdpi.com/article/10.3390/biom14070793/s1, Figure S1: Incorporation of HF datasets; Figure S2: Identification of key MEs in the HF combined dataset through WGCNA; Table S1: Descriptive statistics of the GEO datasets. File S1: Codes for mendelian randomization and bioinformatics analysis in R (version 4.3.1).
