# Supplementary material for: Screening of Secretory Proteins Linking Major Depressive Disorder with Heart Failure Based on Comprehensive Bioinformatics Analysis and Machine Learning
Source: Biomolecules. 2024 Jul 4;14(7):793. doi: 10.3390/biom14070793 (PMC11275063; doi:10.3390/biom14070793)
Supplement: Supplementary file 1 [file biomolecules-14-00793-s001.zip › Supplementary Figures and Tables.pdf]

Supplementary material

Table S1 Descriptive statistics of the GEO datasets

GEO accession	Platform	Origin	Sample(n)		Age(year)		Gender(male)	
			Control	HF	Control	HF	Control	HF
GSE1145	GPL570	Heart	11	15	NA	NA	NA	NA
GSE19303	GPL570	Heart	8	40	43.1±14.7	50.2±9.3	6(75.0%)	28(70.0%)
GSE21610	GPL570	Heart	8	21	35.3±16.5	49.3±13.2	6(75.0%)	18(85.7)
GEO accession	Platform	Origin	Sample		Age		Gender(male)	
			Control	MDD	Control	MDD	Control	MDD
GSE38206	GPL13607	PBMC	9	9	53.8±9.2	57.2±8.5	4(44.4%)	4(44.4%)
GSE80655	GPL11154	AnCg	24	24	50.2±12.5	45.3±13.6	21(87.5%)	18(75.0%)
GSE80655	GPL11154	DLPFC	24	23	50.2±12.5	45.8±13.7	21(87.5%)	17(73.9%)
GSE80655	GPL11154	nAcc	22	22	50.1±12.5	45.0±14.2	20(90.9%)	16(72.7%)

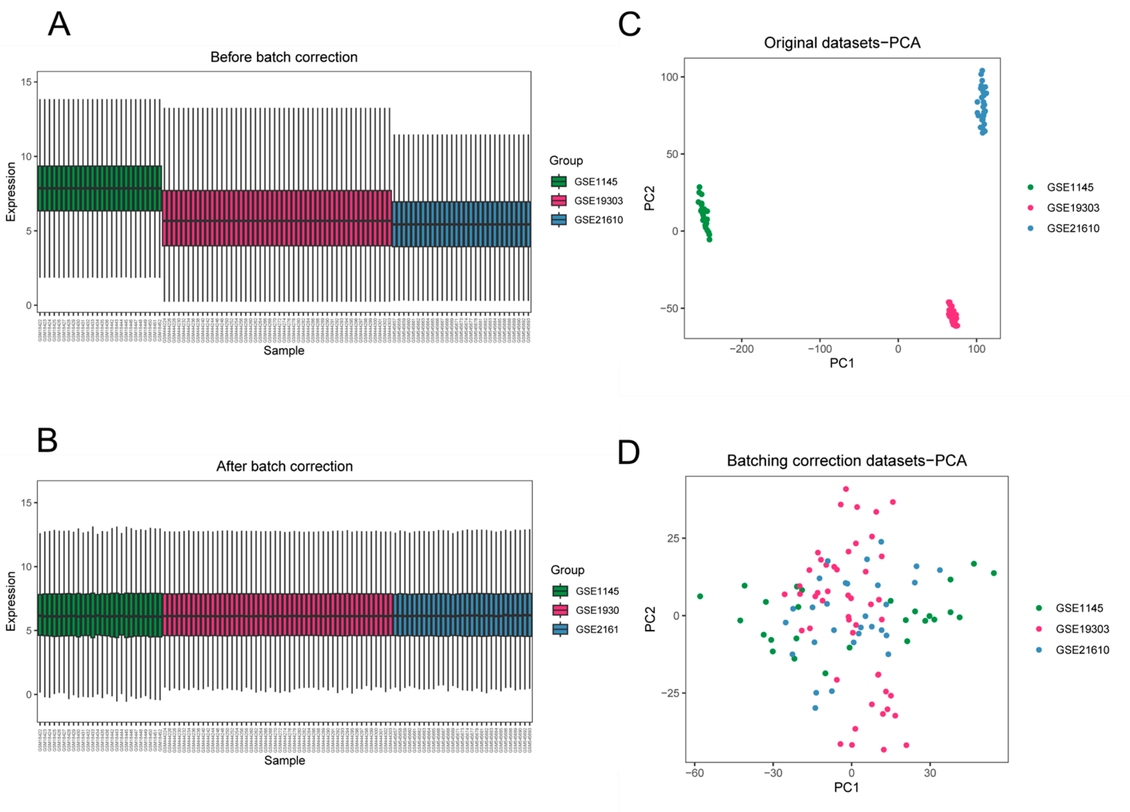

Figure S1. Incorporation of HF datasets. (A, B) Boxplots presenting the distribution of data in the three original HF datasets prior to (A) and following (B) correction of batch effects. (C, D) PCA visualizations demonstrating the relationships among samples in the three original HF datasets prior to (C) and following (D) correction of batch effects. HF, heart failure; PCA, principal component analysis.

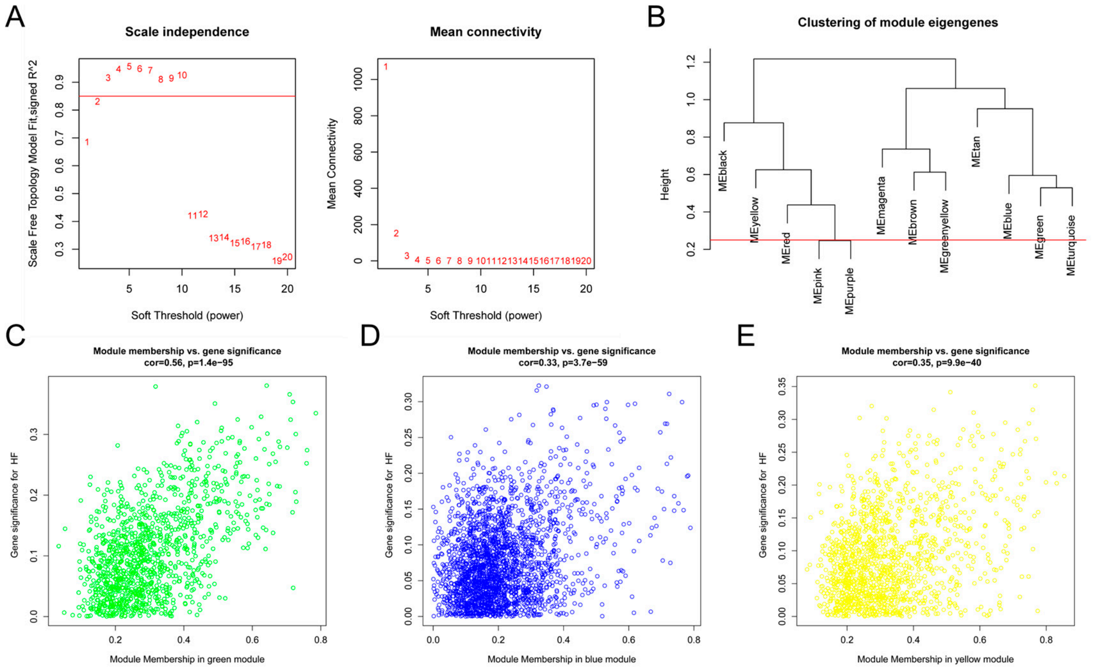

Figure S2. Identification of key MEs in the HF combined dataset through WGCNA. (A) Scale-free topology model for the determination of optimal β value, with β = 3 as the soft threshold based on average connectivity and scale independence. (B) Cluster dendrogram presenting the MEs. (C) The correlation plot between the green module membership and the gene significance of genes in the green module. (D) The correlation plot between the blue module membership and the gene significance of genes in the blue module. (E) The correlation plot between the yellow module membership and the gene significance of genes in the yellow module.
