# Supplementary material for: Screening of Secretory Proteins Linking Major Depressive Disorder with Heart Failure Based on Comprehensive Bioinformatics Analysis and Machine Learning
Source: Biomolecules. 2024 Jul 4;14(7):793. doi: 10.3390/biom14070793 (PMC11275063; doi:10.3390/biom14070793)
Supplement: Supplementary file 1 [file biomolecules-14-00793-s001.zip › Supplementary File S1.pdf]

Codes for mendelian randomization and bioinformatics analysis in R (version 4.3.1)

2.1. MR analysis of the effect of MDD on HF

2.1.1 Instrumental variable selection criteria

```
#p value
install.packages("devtools")
devtools::install_github("mrcieu/gwasglue", force = TRUE)
if (!require("BiocManager", quietly = TRUE))
    install.packages("BiocManager")
BiocManager::install("VariantAnnotation")
install.packages("dplyr")
install.packages("tidyr")
install.packages("CMplot")
library(VariantAnnotation)
library(gwasglue)
library(dplyr)
library(tidyr)
library(CMplot)
inputFile="ieu-a-1188.vcf.gz"
setwd("F:\\ Mendelian\\04.exposure")
vcfRT <- readVcf(inputFile)
data=gwasvcf_to_TwoSampleMR(vcf=vcfRT, type="exposure")
outTab<-subset(data, pval.exposure<5e-06)
write.csv(outTab, file="exposure.pvalue.csv", row.names=F)

#LD
install.packages("remotes")
remotes::install_github("MRCIEU/TwoSampleMR")
library(TwoSampleMR)
library(plinkbinr)
library(ieugwasr)
exposureFile="exposure.pvalue.csv"
setwd("F:\\ Mendelian\\05.LD")
exposure_dat<-read_exposure_data(filename=exposureFile,
```

```
                          sep = ",",
                          snp_col = "SNP",
                          beta_col = "beta.exposure",
                          se_col = "se.exposure",
                          effect_allele_col = "effect_allele.exposure",
                          other_allele_col = "other_allele.exposure",
                          eaf_col = "eaf.exposure",
                          samplesize_col = "samplesize.exposure",
                          clump = F)
exposure_dat_clumped <- clump_data(exposure_dat, clump_kb=10000, clump_r2=0.001)
write.csv(exposure_dat_clumped, file="exposure.LD.csv", row.names=F)

#F
Ffilter=10
inputFile="exposure.LD.csv"
setwd("F:\\Mendelian\\06.F")
dat<-read.csv(inputFile, header=T, sep=",", check.names=F)
N=dat[1,"samplesize.exposure"]
dat=transform(dat,R2=2*((beta.exposure)^2)*eaf.exposure*(1-eaf.exposure))
dat=transform(dat,F=(N-2)*R2/(1-R2))
outTab=dat[dat$F>Ffilter,]
write.csv(dat, "exposure.F.csv", row.names=F)
```

2.1.2 MR and sensitivity analysis

```
library(VariantAnnotation)
library(gwasglue)
library(TwoSampleMR)
exposureName="MDD"
outcomeName="HF"
exposureFile="exposure.F.csv"
outcomeFile=" ebi-a-GCST009541.vcf.gz"
setwd("F:\\Mendelian\\TwoSampleMR")
exposure_dat<-read_exposure_data(filename=exposureFile,
                          sep = ",",
```

```
                                snp_col = "SNP",
                                beta_col = "beta.exposure",
                                se_col = "se.exposure",
                                effect_allele_col = "effect_allele.exposure",
                                other_allele_col = "other_allele.exposure",
                                eaf_col = "eaf.exposure",
                                clump = F)

vcfRT1 <- readVcf(outcomeFile)
outcomeData=gwasvcf_to_TwoSampleMR(vcf=vcfRT1, type="outcome")
outcomeTab<-merge(exposure_dat, outcomeData, by.x="SNP", by.y="SNP")
write.csv(outcomeTab[,-(2:ncol(exposure_dat))], file="outcome.csv")

outcome_dat<-read_outcome_data(snps=exposure_dat$SNP,
                        filename="outcome.csv", sep = ",",
                        snp_col = "SNP",
                        beta_col = "beta.outcome",
                        se_col = "se.outcome",
                        effect_allele_col = "effect_allele.outcome",
                        other_allele_col = "other_allele.outcome",
                        pval_col = "pval.outcome",
                        eaf_col = "eaf.outcome")
exposure_dat$exposure=exposureName
outcome_dat$outcome=outcomeName
dat<-harmonise_data(exposure_dat=exposure_dat,
                            outcome_dat=outcome_dat)
outTab=dat[dat$mr_keep=="TRUE",]
write.csv(outTab, file="table.SNP.csv", row.names=F)
#MR-PRESSO
presso=run_mr_presso(dat)
write.csv(presso[[1]]$`MR-PRESSO results`$`Outlier Test`, file="table.MR-PRESSO.csv")

mrResult=mr(dat)
mrTab=generate_odds_ratios(mrResult)
```

```
write.csv(mrTab, file="table.MRresult.csv", row.names=F)

heterTab=mr_heterogeneity(dat)
write.csv(heterTab, file="table.heterogeneity.csv", row.names=F)

pleioTab=mr_pleiotropy_test(dat)
write.csv(pleioTab, file="table.pleiotropy.csv", row.names=F)

pdf(file="pic.scatter_plot.pdf", width=7.5, height=7)
mr_scatter_plot(mrResult, dat)
dev.off()

res_single=mr_singlesnp(dat)
pdf(file="pic.forest.pdf", width=7, height=6.5)
mr_forest_plot(res_single)
dev.off()

pdf(file="pic.funnel_plot.pdf", width=7, height=6.5)
mr_funnel_plot(singlesnp_results = res_single)
dev.off()

pdf(file="pic.leaveoneout.pdf", width=7, height=6.5)
mr_leaveoneout_plot(leaveoneout_results = mr_leaveoneout(dat))
dev.off()

#forest
pFilter=1
setwd("F:\\ Mendelian\\09.forest")
bioForest=function(inputFile=null, forestFile=null, forestCol=null){
    rt=read.csv(inputFile, header=T, sep=",", check.names=F)
    row.names(rt)=rt$method
    rt=rt[rt$pval<pFilter,]
    method <- rownames(rt)
    or <- sprintf("%.3f",rt$"or")
```

```
    orLow    <- sprintf("%.3f",rt$"or_lci95")
    orHigh <- sprintf("%.3f",rt$"or_uci95")
    OR <- paste0(or,"(",orLow,"-",orHigh,")")
    pVal <- ifelse(rt$pval<0.001, "<0.001", sprintf("%.3f", rt$pval))
    pdf(file=forestFile, width=7, height=4.6)
    n <- nrow(rt)
    nRow <- n+1
    ylim <- c(1,nRow)
    layout(matrix(c(1,2),nc=2),width=c(3.5,2))
    xlim = c(0,3)
    par(mar=c(4,2.5,2,1))
    plot(1,xlim=xlim,ylim=ylim,type="n",axes=F,xlab="",ylab="")
    text.cex=0.8
    text(0,n:1,method,adj=0,cex=text.cex)
    text(1.9,n:1,pVal,adj=1,cex=text.cex);text(1.9,n+1,'pvalue',cex=1,font=2,adj=1)
    text(3.1,n:1,OR,adj=1,cex=text.cex);text(2.7,n+1,'OR',cex=1,font=2,adj=1)
    par(mar=c(4,1,2,1),mgp=c(2,0.5,0))
    xlim                                                                    =
c(min(as.numeric(orLow)*0.975,as.numeric(orHigh)*0.975,0.9),max(as.numeric(orLow),as
.numeric(orHigh))*1.025)
    plot(1,xlim=xlim,ylim=ylim,type="n",axes=F,ylab="",xaxs="i",xlab="OR")
    arrows(as.numeric(orLow),n:1,as.numeric(orHigh),n:1,angle=90,code=3,length=0.05,c
ol="darkblue",lwd=3)
    abline(v=1, col="black", lty=2, lwd=2)
    boxcolor = ifelse(as.numeric(or)>1, forestCol, forestCol)
    points(as.numeric(or), n:1, pch = 15, col = boxcolor, cex=2)
    axis(1)
    dev.off()
}
bioForest(inputFile="table.MRresult.csv", forestFile="table.MRresult.pdf", forestCol="red")
```

2.2. Expression data collection and processing

```
if (!requireNamespace("BiocManager", quietly = TRUE))
install.packages("BiocManager")
```

```
BiocManager::install("limma")
BiocManager::install("sva")

library(limma)
library(sva)
setwd("F:\\HFDP\\Simple\\10.HF_sva")
files=dir()
files=grep("txt$", files, value=T)
geneList=list()

for(file in files){
   if(file=="merge.preNorm.txt"){next}
   if(file=="merge.normalzie.txt"){next}
   rt=read.table(file, header=T, sep="\t", check.names=F)
   geneNames=as.vector(rt[,1])
   uniqGene=unique(geneNames)
   header=unlist(strsplit(file, "\\.|\\-"))
   geneList[[header[1]]]=uniqGene
}
interGenes=Reduce(intersect, geneList)
allTab=data.frame()
batchType=c()
for(i in 1:length(files)){
   inputFile=files[i]
   header=unlist(strsplit(inputFile, "\\.|\\-"))
   rt=read.table(inputFile, header=T, sep="\t", check.names=F)
   rt=as.matrix(rt)
   rownames(rt)=rt[,1]
   exp=rt[,2:ncol(rt)]
   dimnames=list(rownames(exp),colnames(exp))
   data=matrix(as.numeric(as.matrix(exp)),nrow=nrow(exp),dimnames=dimnames)
   rt=avereps(data)
   colnames(rt)=paste0(header[1], "_", colnames(rt))
```

```
    if(i==1){
        allTab=rt[interGenes,]
    }else{
        allTab=cbind(allTab, rt[interGenes,])
    }
    batchType=c(batchType, rep(i,ncol(rt)))
}

outTab=rbind(geneNames=colnames(allTab), allTab)
write.table(outTab, file="merge.preNorm.txt", sep="\t", quote=F, col.names=F)

outTab=ComBat(allTab, batchType, par.prior=TRUE)
outTab=rbind(geneNames=colnames(outTab), outTab)
write.table(outTab, file="merge.normalzie.txt", sep="\t", quote=F, col.names=F)
```

2.3. Differentially expressed genes (DEGs) analysis

```
install.packages("pheatmap")
library(limma)
library(pheatmap)

logFCfilter=0.5
adj.P.Val.Filter=0.05
inputFile="merge.normalzie.txt"
setwd("F:\\HFDP\\Simple\\13.HF_batchDiff\\color")
rt=read.table(inputFile, header=T, sep="\t", check.names=F)
rt=as.matrix(rt)
rownames(rt)=rt[,1]
exp=rt[,2:ncol(rt)]
dimnames=list(rownames(exp),colnames(exp))
data=matrix(as.numeric(as.matrix(exp)),nrow=nrow(exp),dimnames=dimnames)
data=avereps(data)
Type=gsub("(.*)\\_(.*)\\_(.*)", "\\3", colnames(data))
data=data[,order(Type)]
Project=gsub("(.+)\\_(.+)\\_(.+)", "\\1", colnames(data))
```

```
Type=gsub("(.*)\\_(.*)\\_(.*)", "\\3", colnames(data))
colnames(data)=gsub("(.+)\\_(.+)\\_(.+)", "\\2", colnames(data))

design <- model.matrix(~0+factor(Type))
colnames(design) <- c("con","HF")
fit <- lmFit(data,design)
cont.matrix<-makeContrasts(HF-con,levels=design)
fit2 <- contrasts.fit(fit, cont.matrix)
fit2 <- eBayes(fit2)

allDiff=topTable(fit2, adjust='fdr',number=200000)
allDiffOut=rbind(id=colnames(allDiff),allDiff)
write.table(allDiffOut, file="Batch.all.txt", sep="\t", quote=F, col.names=F)

diffSig=allDiff[with(allDiff, (abs(logFC)>logFCfilter & adj.P.Val < adj.P.Val.Filter )), ]
diffSigOut=rbind(id=colnames(diffSig),diffSig)
write.table(diffSigOut, file="Batch.diff.txt", sep="\t", quote=F, col.names=F)
diffGeneExp=data[row.names(diffSig),]
diffGeneExpOut=rbind(id=paste0(colnames(diffGeneExp),"_",Type), diffGeneExp)
write.table(diffGeneExpOut, file="diffGeneExp.txt", sep="\t", quote=F, col.names=F)

geneNum=30
diffSig=diffSig[order(as.numeric(as.vector(diffSig$logFC))),]
diffGeneName=as.vector(rownames(diffSig))
diffLength=length(diffGeneName)
hmGene=c()
if(diffLength>(2*geneNum)){
  hmGene=diffGeneName[c(1:geneNum,(diffLength-geneNum+1):diffLength)]
}else{
  hmGene=diffGeneName
}
hmExp=data[hmGene,]
names(Type)=colnames(data)
Type=as.data.frame(Type)
```

```
Type=cbind(Project, Type)
pdf(file="Batch.heatmap.pdf", width=10, height=7)
pheatmap(hmExp,
         annotation=Type,
         color = colorRampPalette(c("#029149", "white", "#E0367A"))(30),
         cluster_cols =F,
         show_colnames = F,
         scale="row",
         fontsize = 8,
         fontsize_row=6,
         fontsize_col=8)
dev.off()

#install.packages("dplyr")
#install.packages("ggplot2")
#install.packages("ggrepel")
library(dplyr)
library(ggplot2)
library(ggrepel)

files=dir()
files=grep(".all.txt$",files,value=T)

for(inputFile in files){
  rt=read.table(inputFile, header=T, sep="\t", check.names=F)
  Sig=ifelse((rt$adj.P.Val<adj.P.Val.Filter) & (abs(rt$logFC)>logFCfilter),
ifelse(rt$logFC>logFCfilter,"Up","Down"), "None-DEG")
  maxX=ifelse(max(abs(rt$logFC))>10, 10, round(abs(rt$logFC)+0.5))
  rt = mutate(rt, Sig=Sig)
  p = ggplot(rt, aes(logFC, -log10(adj.P.Val)))+
    geom_point(aes(col=Sig))+ xlim(-2, 2)+
    scale_color_manual(values=c("#029149", "gray", "#E0367A"))+
    geom_vline(xintercept=c(-logFCfilter,logFCfilter), col="gray", cex=0.5, linetype=3)+
    geom_hline(yintercept= -log10(adj.P.Val.Filter), col="gray", cex=0.5, linetype=3)+
```

```
        theme_bw()+
        theme(plot.title = element_text(hjust=0.5, size=15, face="bold"),panel.grid = element_blank())+
        labs(title = gsub(".all.txt", "", inputFile))
    outFile=gsub(".all.txt", ".vol.pdf", inputFile)
    pdf(file=outFile, width=6, height=5.5)
    print(p)
    dev.off()
}
```

2.4. WGCNA

```
BiocManager::install(c("GO.db", "preprocessCore", "impute","limma"))
install.packages(c("matrixStats", "Hmisc", "foreach", "doParallel", "fastcluster", "dynamicTreeCut", "survival"))
install.packages("WGCNA")
library(limma)
library(WGCNA)
setwd("F:\\HFDP\\Simple\\15.WGCNA")
expFile="merge.normalzie.txt"

rt=read.table(expFile, header=T, sep="\t", check.names=F)
rt=as.matrix(rt)
rownames(rt)=rt[,1]
exp=rt[,2:ncol(rt)]
dimnames=list(rownames(exp),colnames(exp))
data=matrix(as.numeric(as.matrix(exp)),nrow=nrow(exp),dimnames=dimnames)
data=avereps(data)
data=data[apply(data,1,sd)>0,]
Type=gsub("(.*)\\_(.*)", "\\2", colnames(data))
conCount=length(Type[Type=="con"])
treatCount=length(Type[Type=="HF"])
datExpr0=t(data)
gsg = goodSamplesGenes(datExpr0, verbose = 3)
if (!gsg$allOK)
```

```
{
   # Optionally, print the gene and sample names that were removed:
   if (sum(!gsg$goodGenes)>0)
      printFlush(paste("Removing genes:", paste(names(datExpr0)[!gsg$goodGenes],
collapse = ", ")))
   if (sum(!gsg$goodSamples)>0)
      printFlush(paste("Removing samples:",
paste(rownames(datExpr0)[!gsg$goodSamples], collapse = ", ")))
   # Remove the offending genes and samples from the data:
   datExpr0 = datExpr0[gsg$goodSamples, gsg$goodGenes]
}

sampleTree = hclust(dist(datExpr0), method = "average")
pdf(file = "1_sample_cluster.pdf", width = 12, height = 9)
par(cex = 0.6)
par(mar = c(0,4,2,0))
plot(sampleTree, main = "Sample clustering to detect outliers", sub="", xlab="", cex.lab = 1.5,
cex.axis = 1.5, cex.main = 2)
abline(h = 20000, col = "red")
dev.off()

clust = cutreeStatic(sampleTree, cutHeight = 20000, minSize = 10)
table(clust)
keepSamples = (clust==1)
datExpr0 = datExpr0[keepSamples, ]


traitData=data.frame(Con=c(rep(1,conCount),rep(0,treatCount)),
                     Treat=c(rep(0,conCount),rep(1,treatCount)))
row.names(traitData)=colnames(data)
fpkmSamples = rownames(datExpr0)
traitSamples =rownames(traitData)
sameSample=intersect(fpkmSamples,traitSamples)
datExpr0=datExpr0[sameSample,]
```

```
datTraits=traitData[sameSample,]

sampleTree2 = hclust(dist(datExpr0), method = "average")
traitColors = numbers2colors(datTraits, signed = FALSE)
pdf(file="2_sample_heatmap.pdf",width=12,height=12)
plotDendroAndColors(sampleTree2, traitColors,
                    groupLabels = names(datTraits),
                    main = "Sample dendrogram and trait heatmap")
dev.off()

enableWGCNAThreads()
powers = c(1:20)
sft = pickSoftThreshold(datExpr0, powerVector = powers, verbose = 5)
pdf(file="3_scale_independence.pdf",width=9,height=5)
par(mfrow = c(1,2))
cex1 = 0.85
plot(sft$fitIndices[,1], -sign(sft$fitIndices[,3])*sft$fitIndices[,2],
     xlab="Soft Threshold (power)",ylab="Scale Free Topology Model Fit,signed
R^2",type="n",
     main = paste("Scale independence"));
text(sft$fitIndices[,1], -sign(sft$fitIndices[,3])*sft$fitIndices[,2],
     labels=powers,cex=cex1,col="red");
abline(h=0.85,col="red")
plot(sft$fitIndices[,1], sft$fitIndices[,5],
     xlab="Soft Threshold (power)",ylab="Mean Connectivity", type="n",
     main = paste("Mean connectivity"))
text(sft$fitIndices[,1], sft$fitIndices[,5], labels=powers, cex=cex1,col="red")
dev.off()
sft
softPower =sft$powerEstimate
adjacency = adjacency(datExpr0, power = softPower)
softPower

pdf(file="3_softConnectivity.pdf", width=9, height=5)
```

```
k <- softConnectivity(datE=datExpr0,power=softPower)
#sizeGrWindow(10, 5)
par(mfrow=c(1,2))
hist(k)
scaleFreePlot(k,main="Check Scale free topology\n")
dev.off()

TOM = TOMsimilarity(adjacency)
dissTOM = 1-TOM

geneTree = hclust(as.dist(dissTOM), method = "average");
pdf(file="4_gene_clustering.pdf",width=12,height=9)
plot(geneTree, xlab="", sub="", main = "Gene clustering on TOM-based dissimilarity",
     labels = FALSE, hang = 0.04)
dev.off()
minModuleSize = 60
dynamicMods = cutreeDynamic(dendro = geneTree, distM = dissTOM,
                            deepSplit = 2, pamRespectsDendro = FALSE,
                            minClusterSize = minModuleSize);
table(dynamicMods)
dynamicColors = labels2colors(dynamicMods)
table(dynamicColors)
pdf(file="5_Dynamic_Tree.pdf",width=8,height=6)
plotDendroAndColors(geneTree, dynamicColors, "Dynamic Tree Cut",
                    dendroLabels = FALSE, hang = 0.03,
                    addGuide = TRUE, guideHang = 0.05,
                    main = "Gene dendrogram and module colors")
dev.off()

MEList = moduleEigengenes(datExpr0, colors = dynamicColors)
MEs = MEList$eigengenes
MEDiss = 1-cor(MEs);
METree = hclust(as.dist(MEDiss), method = "average")
pdf(file="6_Clustering_module.pdf",width=7,height=6)
```

```
plot(METree, main = "Clustering of module eigengenes",
     xlab = "", sub = "")
MEDissThres = 0.25
abline(h=MEDissThres, col = "red")
dev.off()
merge = mergeCloseModules(datExpr0, dynamicColors, cutHeight = MEDissThres,
verbose = 3)
mergedColors = merge$colors
mergedMEs = merge$newMEs
pdf(file="7_merged_dynamic.pdf", width = 9, height = 6)
plotDendroAndColors(geneTree, mergedColors,"Dynamic Tree Cut",
                    dendroLabels = FALSE, hang = 0.03,
                    addGuide = TRUE, guideHang = 0.05,
                    main = "Gene dendrogram and module colors")
dev.off()
moduleColors = mergedColors
table(moduleColors)
colorOrder = c("grey", standardColors(50))
moduleLabels = match(moduleColors, colorOrder)-1
MEs = mergedMEs

nGenes = ncol(datExpr0)
nSamples = nrow(datExpr0)
moduleTraitCor = cor(MEs, datTraits, use = "p")
moduleTraitPvalue = corPvalueStudent(moduleTraitCor, nSamples)
pdf(file="8_Module_trait.pdf", width=6, height=5.5)
textMatrix = paste(signif(moduleTraitCor, 2), "\n(",
                   signif(moduleTraitPvalue, 1), ")", sep = "")
dim(textMatrix) = dim(moduleTraitCor)
par(mar = c(5, 10, 3, 3))
labeledHeatmap(Matrix = moduleTraitCor,
               xLabels = names(datTraits),
               yLabels = names(MEs),
               ySymbols = names(MEs),
```

```
                       colorLabels = FALSE,
                       colors = blueWhiteRed(50),
                       textMatrix = textMatrix,
                       setStdMargins = FALSE,
                       cex.text = 0.5,
                       zlim = c(-1,1),
                       main = paste("Module-trait relationships"))
dev.off()

modNames = substring(names(MEs), 3)
geneModuleMembership = as.data.frame(cor(datExpr0, MEs, use = "p"))
MMPvalue = as.data.frame(corPvalueStudent(as.matrix(geneModuleMembership),
nSamples))
names(geneModuleMembership) = paste("MM", modNames, sep="")
names(MMPvalue) = paste("p.MM", modNames, sep="")
traitNames=names(datTraits)
geneTraitSignificance = as.data.frame(cor(datExpr0, datTraits, use = "p"))
GSPvalue = as.data.frame(corPvalueStudent(as.matrix(geneTraitSignificance), nSamples))
names(geneTraitSignificance) = paste("GS.", traitNames, sep="")
names(GSPvalue) = paste("p.GS.", traitNames, sep="")

y=datTraits[,1]
GS1=as.numeric(cor(y, datExpr0, use="p"))
GeneSignificance=abs(GS1)
ModuleSignificance=tapply(GeneSignificance, mergedColors, mean, na.rm=T)
pdf(file="9_GeneSignificance.pdf", width=11, height=7)
plotModuleSignificance(GeneSignificance, mergedColors)
dev.off()

trait="Treat"
traitColumn=match(trait,traitNames)
for (module in modNames){
   column = match(module, modNames)
   moduleGenes = moduleColors==module
```

```
    if (nrow(geneModuleMembership[moduleGenes,]) > 1){
        outPdf=paste("10_", trait, "_", module,".pdf",sep="")
        pdf(file=outPdf,width=7,height=7)
        par(mfrow = c(1,1))
        verboseScatterplot(abs(geneModuleMembership[moduleGenes, column]),
                               abs(geneTraitSignificance[moduleGenes, traitColumn]),
                               xlab = paste("Module Membership in", module, "module"),
                               ylab = paste("Gene significance for ",trait),
                               main = paste("Module membership vs. gene significance\n"),
                               cex.main = 1.2, cex.lab = 1.2, cex.axis = 1.2, col = module)
        abline(v=0.8,h=0.5,col="red")
        dev.off()
    }
}

probes = colnames(datExpr0)
geneInfo0 = data.frame(probes= probes,
                               moduleColor = moduleColors)
for (Tra in 1:ncol(geneTraitSignificance))
{
    oldNames = names(geneInfo0)
    geneInfo0 = data.frame(geneInfo0, geneTraitSignificance[,Tra],
                               GSPvalue[, Tra])
    names(geneInfo0) = c(oldNames,names(geneTraitSignificance)[Tra],
                               names(GSPvalue)[Tra])
}

for (mod in 1:ncol(geneModuleMembership))
{
    oldNames = names(geneInfo0)
    geneInfo0 = data.frame(geneInfo0, geneModuleMembership[,mod],
                               MMPvalue[, mod])
    names(geneInfo0) = c(oldNames,names(geneModuleMembership)[mod],
                               names(MMPvalue)[mod])
```

```
}
geneOrder =order(geneInfo0$moduleColor)
geneInfo = geneInfo0[geneOrder, ]
write.table(geneInfo, file = "GS_MM.xls",sep="\t",row.names=F)
for (mod in 1:nrow(table(moduleColors)))
{
  modules = names(table(moduleColors))[mod]
  probes = colnames(datExpr0)
  inModule = (moduleColors == modules)
  modGenes = probes[inModule]
  write.table(modGenes, file =paste0("module_",modules,".txt"),sep="\t",row.names=F,col.names=F,quote=F)
}
```

2.9. ML algorithms

2.9.1 Venn

```
install.packages("venn")
library(venn)
outFile="interGenes.txt"
setwd("F:\\HFDP\\Simple\\24.dia_venn")
geneList=list()
rt=read.table("Batch.diff.up.txt",header=T,sep="\t",check.names=F)
geneNames=as.vector(rt[,1])
geneNames=gsub("^ | $","",geneNames)
uniqGene=unique(geneNames)
geneList[["HF DEGs_up"]]=uniqGene
rt=read.table("DP4_UNION_GENES.txt",header=T,sep="\t",check.names=F)
geneNames=as.vector(rt[,1])
geneNames=gsub("^ | $","",geneNames)
uniqGene=unique(geneNames)
geneList[["MDD secreted proteins"]]=uniqGene
mycol=c("#029149","#E0367A","#5D90BA","#431A3D","#FFD121","#D8D155","#223D6C","#D20A13","#088247","#11AA4D","#7A142C","#5D90BA","#64495D","#7CC767")
pdf(file="interGenes.pdf", width=5, height=5)
```

```
venn(geneList,col=mycol[1:length(geneList)],zcolor=mycol[1:length(geneList)],box=F,ilab
els=F)
dev.off()
interGenes=Reduce(intersect,geneList)
write.table(file=outFile,interGenes,sep="\t",quote=F,col.names=F,row.names=F)
```

2.9.2. RF/SVM/XGB/GLM algorithms

```
install.packages("caret")
install.packages("DALEX")
install.packages("randomForest")
install.packages("kernlab")
install.packages("pROC")
install.packages("xgboost")
library(caret)
library(DALEX)
library(ggplot2)
library(randomForest)
library(kernlab)
library(xgboost)
library(pROC)
set.seed(1234)
inputFile="merge.normalzie.txt"
geneFile="interGenes.txt"
setwd("F:\\HFDP\\Simple\\25.model")
data=read.table(inputFile, header=T, sep="\t", check.names=F, row.names=1)
geneRT=read.table(geneFile, header=F, sep="\t", check.names=F)
data=data[as.vector(geneRT[,1]),]
row.names(data)=gsub("-", "_", row.names(data))

data=t(data)
group=gsub("(.*)\\_(.*)", "\\2", row.names(data))
data=as.data.frame(data)
data$Type=group
inTrain<-createDataPartition(y=data$Type, p=0.5, list=F)
```

```
train<-data[inTrain,]
test<-data[-inTrain,]
#RF
control=trainControl(method="repeatedcv", number=5, savePredictions=TRUE)
mod_rf = train(Type ~ ., data = train, method='rf', trControl = control)
#SVM
mod_svm=train(Type ~., data = train, method = "svmRadial", prob.model=TRUE, trControl=control)
#XGB
mod_xgb=train(Type ~., data = train, method = "xgbDART", trControl=control)
#GLM
mod_glm=train(Type ~., data = train, method = "glm", family="binomial", trControl=control)

p_fun=function(object, newdata){
  predict(object, newdata=newdata, type="prob")[,2]
}
yTest=ifelse(test$Type=="con", 0, 1)
#RF
explainer_rf=explain(mod_rf, label = "RF",
                     data = test, y = yTest,
                     predict_function = p_fun,
                     verbose = FALSE)
mp_rf=model_performance(explainer_rf)
#SVM
explainer_svm=explain(mod_svm, label = "SVM",
                      data = test, y = yTest,
                      predict_function = p_fun,
                      verbose = FALSE)
mp_svm=model_performance(explainer_svm)
#XGB
explainer_xgb=explain(mod_xgb, label = "XGB",
                      data = test, y = yTest,
                      predict_function = p_fun,
```

```
                                verbose = FALSE)
mp_xgb=model_performance(explainer_xgb)
#GLM
explainer_glm=explain(mod_glm, label = "GLM",
                                data = test, y = yTest,
                                predict_function = p_fun,
                                verbose = FALSE)
mp_glm=model_performance(explainer_glm)

pdf(file="residual.pdf", width=6, height=6)
p1 <- plot(mp_rf, mp_svm, mp_xgb, mp_glm)
print(p1)
dev.off()

pdf(file="boxplot.pdf", width=6, height=6)
p2 <- plot(mp_rf, mp_svm, mp_xgb, mp_glm, geom = "boxplot")
print(p2)
dev.off()

pred1=predict(mod_rf, newdata=test, type="prob")
pred2=predict(mod_svm, newdata=test, type="prob")
pred3=predict(mod_xgb, newdata=test, type="prob")
pred4=predict(mod_glm, newdata=test, type="prob")
roc1=roc(yTest, as.numeric(pred1[,2]))
roc2=roc(yTest, as.numeric(pred2[,2]))
roc3=roc(yTest, as.numeric(pred3[,2]))
roc4=roc(yTest, as.numeric(pred4[,2]))
pdf(file="ROC.pdf", width=5, height=5)
plot(roc1, print.auc=F, legacy.axes=T, main="", col="red")
plot(roc2, print.auc=F, legacy.axes=T, main="", col="blue", add=T)
plot(roc3, print.auc=F, legacy.axes=T, main="", col="green", add=T)
plot(roc4, print.auc=F, legacy.axes=T, main="", col="yellow", add=T)
legend('bottomright',
          c(paste0('RF: ',sprintf("%.03f",roc1$auc)),
```

```
        paste0('SVM: ',sprintf("%.03f",roc2$auc)),
        paste0('XGB: ',sprintf("%.03f",roc3$auc)),
        paste0('GLM: ',sprintf("%.03f",roc4$auc))),
      col=c("red","blue","green","yellow"), lwd=2, bty = 'n')
dev.off()
importance_rf<-variable_importance(
  explainer_rf,
  loss_function = loss_root_mean_square
)
importance_svm<-variable_importance(
  explainer_svm,
  loss_function = loss_root_mean_square
)
importance_glm<-variable_importance(
  explainer_glm,
  loss_function = loss_root_mean_square
)
importance_xgb<-variable_importance(
  explainer_xgb,
  loss_function = loss_root_mean_square
)
pdf(file="importance.pdf", width=7, height=10)
plot(importance_rf[c(1,(ncol(data)-8):(ncol(data)+1)),],
     importance_svm[c(1,(ncol(data)-8):(ncol(data)+1)),],
     importance_xgb[c(1,(ncol(data)-8):(ncol(data)+1)),],
     importance_glm[c(1,(ncol(data)-8):(ncol(data)+1)),])
dev.off()
geneNum=8
write.table(importance_rf[(ncol(data)-geneNum+2):(ncol(data)+1),],
file="importanceGene.RF.txt", sep="\t", quote=F, row.names=F)
write.table(importance_svm[(ncol(data)-geneNum+2):(ncol(data)+1),],
file="importanceGene.SVM.txt", sep="\t", quote=F, row.names=F)
write.table(importance_xgb[(ncol(data)-geneNum+2):(ncol(data)+1),],
file="importanceGene.XGB.txt", sep="\t", quote=F, row.names=F)
```

```
write.table(importance_glm[(ncol(data)-geneNum+2):(ncol(data)+1),],
file="importanceGene.GLM.txt", sep="\t", quote=F, row.names=F)
write.table(data, file="HF_status.txt", sep="\t", quote=F, row.names=T)
```

2.10. The construction of nomogram and the assessment of diagnostic marker prediction model

```
install.packages("rms")
install.packages("rmda")
library(rms)
library(rmda)

inputFile="merge.normalzie.txt"
geneFile="interGenes_mechine_learning.txt"
setwd("F:\\HFDP\\Simple\\28.Nomo")
data=read.table(inputFile, header=T, sep="\t", check.names=F, row.names=1)
row.names(data)=gsub("-", "_", row.names(data))
geneRT=read.table(geneFile, header=F, sep="\t", check.names=F)
data=data[as.vector(geneRT[,1]),]
data=t(data)
group=gsub("(.*)\\_(.*)", "\\2", row.names(data))
rt=cbind(as.data.frame(data), Type=group)
paste(colnames(data), collapse="+")
ddist=datadist(rt)
options(datadist="ddist")

lrmModel=lrm(Type~ ISLR+SFRP4, data=rt, x=T, y=T)
nomo=nomogram(lrmModel, fun=plogis,
              fun.at=c(0.0001,0.1,0.3,0.5,0.7,0.9,0.99),
              lp=F, funlabel="Risk of Disease")
pdf("Nomo.pdf", width=10, height=6)
plot(nomo)
dev.off()
cali=calibrate(lrmModel, method="boot", B=1000)
pdf("Calibration.pdf", width=5.5, height=5.5)
```

```
plot(cali,
     xlab="Predicted probability",
     ylab="Actual probability", sub=F)
dev.off()
rt$Type=ifelse(rt$Type=="con", 0, 1)
dc=decision_curve(Type ~ ISLR+SFRP4, data=rt,
                  family = binomial(link ='logit'),
                  thresholds= seq(0,1,by = 0.01),
                  confidence.intervals = 0.95)
pdf(file="DCA.pdf", width=5.5, height=5.5)
plot_decision_curve(dc,
                    curve.names="Model",
                    xlab="Threshold probability",
                    cost.benefit.axis=T,
                    col="red",
                    confidence.intervals=FALSE,
                    standardize=FALSE)
dev.off()

library(glmnet)
library(pROC)
expFile="merge.normalzie.txt"
geneFile="interGenes_mechine_learning.txt"
setwd("F:\\HFDP\\Simple\\29.ROC")

rt=read.table(expFile, header=T, sep="\t", check.names=F, row.names=1)
y=gsub("(.*)\\_(.*)", "\\2", colnames(rt))
y=ifelse(y=="con", 0, 1)

geneRT=read.table(geneFile, header=F, sep="\t", check.names=F)

bioCol=rainbow(nrow(geneRT), s=0.9, v=0.9)
aucText=c()
k=0
```

```
for(x in as.vector(geneRT[,1])){
   k=k+1
   roc1=roc(y, as.numeric(rt[x,]))
   if(k==1){
      pdf(file="ROC.genes.pdf", width=5, height=4.75)
      plot(roc1, print.auc=F, col=bioCol[k], legacy.axes=T, main="")
      aucText=c(aucText, paste0(x,", AUC=",sprintf("%.3f",roc1$auc[1])))
   }else{
      plot(roc1, print.auc=F, col=bioCol[k], legacy.axes=T, main="", add=TRUE)
      aucText=c(aucText, paste0(x,", AUC=",sprintf("%.3f",roc1$auc[1])))
   }
}
legend("bottomright", aucText, lwd=2, bty="n", col=bioCol[1:(ncol(rt)-1)])
dev.off()

rt=rt[as.vector(geneRT[,1]),]
rt=as.data.frame(t(rt))
logit=glm(y ~ ., family=binomial(link='logit'), data=rt)
pred=predict(logit, newx=rt)

roc1=roc(y, as.numeric(pred))
ci1=ci.auc(roc1, method="bootstrap")
ciVec=as.numeric(ci1)
pdf(file="ROC.model.pdf", width=5, height=4.75)
plot(roc1, print.auc=TRUE, col="red", legacy.axes=T, main="Model")
text(0.39, 0.43, paste0("95% CI: ",sprintf("%.03f",ciVec[1]),"-",sprintf("%.03f",ciVec[3])), col="red")
dev.off()
```

2.11. Immune cell infiltration measurement

```
library(devtools)
install_github('ebecht/MCPcounter',ref='master', subdir='Source')
library(limma)
library(MCPcounter)
```

```
expFile="merge.normalzie.txt"
setwd("F:\\HFDP\\Simple\\39.MCPcounter")
rt=read.table(expFile, header=T, sep="\t", check.names=F)
rt=as.matrix(rt)
rownames(rt)=rt[,1]
exp=rt[,2:ncol(rt)]
dimnames=list(rownames(exp),colnames(exp))
mat=matrix(as.numeric(as.matrix(exp)),nrow=nrow(exp),dimnames=dimnames)
mat=avereps(mat)
data=mat[rowMeans(mat)>0,]

MCPcounter.estimate=MCPcounter.estimate(data,
                                        featuresType="HUGO_symbols",

probesets=read.table("probesets.txt",sep="\t",stringsAsFactors=FALSE,colClasses="charac
ter"),

genes=read.table("genesets.txt",sep="\t",stringsAsFactors=FALSE,header=TRUE,colClass
es="character",check.names=FALSE)
)
out=rbind(ID=colnames(MCPcounter.estimate), MCPcounter.estimate)
write.table(out, file="MCPcounter.result.txt", sep="\t", quote=F, col.names=F)

install.packages("corrplot")
library(pheatmap)
library(corrplot)
inputFile="MCPcounter.result.txt"
setwd("F:\\HFDP\\Simple\\41.cellCor")

rt=read.table(inputFile, header=T, sep="\t", check.names=F, row.names=1)
rt=t(rt)
con=grepl("_con", rownames(rt), ignore.case=T)
treat=grepl("_HF", rownames(rt), ignore.case=T)
```

```
conData=rt[con,]
treatData=rt[treat,]
conNum=nrow(conData)
treatNum=nrow(treatData)
data=t(rbind(conData,treatData))

Type=c(rep("con",conNum), rep("HF",treatNum))
names(Type)=colnames(data)
Type=as.data.frame(Type)
pdf(file="heatmap.pdf", width=7, height=4)
pheatmap(data,
         annotation=Type,
         color=colorRampPalette(c("#029149", "white", "#E0367A"))(30),
         cluster_cols=F,
         show_colnames=F,
         scale="row",
         fontsize = 7,
         fontsize_row=7,
         fontsize_col=7)
dev.off()

pdf("corHeatmap.pdf", width=12, height=12)
corrplot(corr=cor(treatData),
         method = "color",
         order = "hclust",
         tl.col="black",
         addCoef.col = "black",
         number.cex = 0.8,
         col=colorRampPalette(c("#029149", "white", "#E0367A"))(30),
)
dev.off()


library(limma)
```

```
library(reshape2)
library(tidyverse)
library(ggplot2)
expFile="interGenesGeneExp.txt"
immFile="MCPcounter.result.txt"
setwd("F:\\HFDP\\Simple\\41.cellCor")

rt=read.table(expFile, header=T, sep="\t", check.names=F)
rt=as.matrix(rt)
rownames(rt)=rt[,1]
exp=rt[,2:ncol(rt)]
dimnames=list(rownames(exp),colnames(exp))
data=matrix(as.numeric(as.matrix(exp)),nrow=nrow(exp),dimnames=dimnames)
data=avereps(data)
data=t(data)

immune=read.table(immFile, header=T, sep="\t", check.names=F, row.names=1)
immune=t(immune)
sameSample=intersect(row.names(data), row.names(immune))
data=data[sameSample,,drop=F]
immune=immune[sameSample,,drop=F]

outTab=data.frame()
for(cell in colnames(immune)){
  if(sd(immune[,cell])==0){next}
  for(gene in colnames(data)){
    x=as.numeric(immune[,cell])
    y=as.numeric(data[,gene])
    corT=cor.test(x,y,method="spearman")
    cor=corT$estimate
    pvalue=corT$p.value
    text=ifelse(pvalue<0.001,"***",ifelse(pvalue<0.01,"**",ifelse(pvalue<0.05,"*","")))
    outTab=rbind(outTab,cbind(Gene=gene, Immune=cell, cor, text, pvalue))
  }
```

```
}

outTab$cor=as.numeric(outTab$cor)
pdf(file="cor.pdf", width=6, height=4)
ggplot(outTab, aes(Immune, Gene)) +
   geom_tile(aes(fill = cor), colour = "grey", size = 1)+
   scale_fill_gradient2(low = "#029149", mid = "white", high = "#E0367A") +
   geom_text(aes(label=text),col ="black",size = 3) +
   theme_minimal() +
   theme(axis.title.x=element_blank(),                              axis.ticks.x=element_blank(),
axis.title.y=element_blank(),
            axis.text.x = element_text(angle = 45, hjust = 1, size = 8, face = "bold"),
            axis.text.y = element_text(size = 8, face = "bold")) +
   labs(fill =paste0("*** p<0.001","\n", "** p<0.01","\n", " * p<0.05","\n", "\n","Correlation"))
+
   scale_x_discrete(position = "bottom")
dev.off()
```
